# Supplementary material for: IonBench: A benchmark of optimisation strategies for mathematical models of ion channel currents
Source: PLoS Comput Biol. 2025 Aug 14;21(8):e1013319. doi: 10.1371/journal.pcbi.1013319 (PMC12373289; doi:10.1371/journal.pcbi.1013319)
Supplement: S1 Text — A: Problem definitions. A detailed description of the problems implemented in ionBench, including the protocols, data, parameter sampling methods and simulation settings. B: Model solve time. A comparison of model solve times for different approaches. C: Significance. Pseudocode implementations of the ERT bootstrapping algorithm and further method details and results for testing significance. D: Profile likelihood. Profile likelihood plots for each of the five problems. E: Performance of all approaches. Reproductions of Fig 7 showing the performance of all approach-problem pairings on their best run. (PDF) [file pcbi.1013319.s001.pdf]

A Problem definitions

A.1 Problem data

All of the problems require generating synthetic data for the optimisation. For the Staircase and Loewe problems, these data are simulated current traces. These current traces are recorded at 2 kHz, while the Moreno I_{Na} problem has varying frequencies to record its summary statistics. Additionally, the Staircase problems have i.i.d. $\sim \mathcal{N}(0, \sigma)$ noise added over the top of the current trace. The σ for the noise is calculated as $\sigma = \frac{0.05}{n} \sum_{i=1}^{n} |I(t_i)|$, where $I(t_i)$ is the current at time step t_i.

A.2 Cost function

All of the problems use RMSE (Root Mean Squared Error) cost functions.

For the Moreno I_{Na} problem, this is a weighted RMSE of the summary statistic curves. The error for each data point is weighted based on how many data points are in that curve. This means the final RMSE evenly weights errors in each of the summary statistics, rather than being biased towards the one with the most data points.

A.3 Parameters

The true parameters for each of the problems and the parameter bounds/sampling regions for the Staircase HH, MM, Loewe I_{Kr}, I_{Kur} and Moreno I_{Na} problems are given in Tables A, B, C, D, and E, respectively.

One thing to note for the Loewe problem rates, the specification of the model indicates the 'rates' only influence the timescale of the states, but not the steady state. The steady state is determined using separate, independent parameters.

Table A. Staircase HH parameters.

Index	True value	Bounds	Log-transformable
1	$2.26 \times 10^{-4}\,\text{ms}^{-1}$	$(10^{-7}, 10^{3})$	Yes
2	$6.99 \times 10^{-2}\,\text{mV}^{-1}$	$(10^{-7}, 0.4)$	No
3	$3.45 \times 10^{-5}\,\text{ms}^{-1}$	$(10^{-7}, 10^{3})$	Yes
4	$5.462 \times 10^{-2}\,\text{mV}^{-1}$	$(10^{-7}, 0.4)$	No
5	$8.73 \times 10^{-2}\,\text{ms}^{-1}$	$(10^{-7}, 10^{3})$	Yes
6	$8.91 \times 10^{-3}\,\text{mV}^{-1}$	$(10^{-7}, 0.4)$	No
7	$5.15 \times 10^{-3}\,\text{ms}^{-1}$	$(10^{-7}, 10^{3})$	Yes
8	$3.158 \times 10^{-2}\,\text{mV}^{-1}$	$(10^{-7}, 0.4)$	No
9	$0.1524\,\mu\text{S}$	$(0.02, 0.2)$	No

The indices match those in Fig 4. True values describe the parameters used to generate the data. Bounds are described as upper and lower bounds on parameters and define the parameter sampling region. The Staircase problems also ensure rate bounds are satisfied for sampled parameters. Log-transformable describes which parameters are transformed when log transforms are enabled.

A.4 Initial conditions

All problems are solved from steady state. This is solved both by `ionBench` and by `myokit`. The `ionBench` initial conditions are set up to allow automatic differentiation of the initial conditions with respect to the parameters, which is used to set the initial conditions of the sensitivity curves.

The implementation in `ionBench` does not include the same checks that are given in `myokit`, such as verifying the steady state is stable (which may be violated for some parameter combinations). This is why we also calculate the steady state using `myokit`. If there is any disagreement between `ionBench` and `myokit` in the steady state, then we instead simulate starting from a fully closed initial condition.

Table B. Staircase MM parameters.

Index	True value	Bounds	Log-transformable
1	$2.0618 \times 10^{-1}\,\mathrm{ms}^{-1}$	$(10^{-7}, 10^{3})$	Yes
2	$1.12 \times 10^{-2}\,\mathrm{mV}^{-1}$	$(10^{-7}, 0.4)$	No
3	$4.209 \times 10^{-2}\,\mathrm{ms}^{-1}$	$(10^{-7}, 10^{3})$	Yes
4	$2.202 \times 10^{-2}\,\mathrm{ms}^{-1}$	$(10^{-7}, 10^{3})$	Yes
5	$3.65 \times 10^{-2}\,\mathrm{mV}^{-1}$	$(10^{-7}, 0.4)$	No
6	$4.1811 \times 10^{-1}\,\mathrm{ms}^{-1}$	$(10^{-7}, 10^{3})$	Yes
7	$2.23 \times 10^{-2}\,\mathrm{mV}^{-1}$	$(10^{-7}, 0.4)$	No
8	$1.3279 \times 10^{-1}\,\mathrm{ms}^{-1}$	$(10^{-7}, 10^{3})$	Yes
9	$6.03 \times 10^{-2}\,\mathrm{mV}^{-1}$	$(10^{-7}, 0.4)$	No
10	$8.094 \times 10^{-2}\,\mathrm{ms}^{-1}$	$(10^{-7}, 10^{3})$	Yes
11	$2.262 \times 10^{-4}\,\mathrm{ms}^{-1}$	$(10^{-7}, 10^{3})$	Yes
12	$3.99 \times 10^{-2}\,\mathrm{mV}^{-1}$	$(10^{-7}, 0.4)$	No
13	$4.15 \times 10^{-2}\,\mathrm{ms}^{-1}$	$(10^{-7}, 10^{3})$	Yes
14	$3.12 \times 10^{-2}\,\mathrm{mV}^{-1}$	$(10^{-7}, 0.4)$	No
15	$0.024\,\mu\mathrm{S}$	$(0.02, 0.2)$	No

The indices match those in Fig 4. True values describe the parameters used to generate the data. Bounds are described as upper and lower bounds on parameters and define the parameter sampling region. The Staircase problems also ensure rate bounds are satisfied for sampled parameters. Log-transformable describes which parameters are transformed when log transforms are enabled.

Table C. Loewe $\mathbf{I_{Kr}}$ parameters.

Index	True value	Bounds	Log-transformable
1	$3 \times 10^{-4}\,\mathrm{ms}^{-1}\,\mathrm{mV}^{-1}$	$(3 \times 10^{-5}, 3 \times 10^{-3})$	Yes
2	$14.1\,\mathrm{mV}$	$(-45.9, 74.1)$	No
3	$5\,\mathrm{mV}$	$(0.5, 50)$	Yes
4	$3.3328\,\mathrm{mV}$	$(-56.6672, 63.3328)$	No
5	$5.1237\,\mathrm{mV}$	$(0.51237, 51.237)$	Yes
6	1	$(0.1, 10)$	Yes
7	$14.1\,\mathrm{mV}$	$(-45.9, 74.1)$	No
8	$6.5\,\mathrm{mV}$	$(0.65, 65)$	Yes
9	$15\,\mathrm{mV}$	$(-45, 75)$	No
10	$22.4\,\mathrm{mV}$	$(2.24, 224)$	Yes
11	$2.9411765 \times 10^{-2}\,\mathrm{nS}\,\mathrm{pF}^{-1}$	$(2.9411765 \times 10^{-3}, 2.9411765 \times 10^{-1})$	Yes
12	$138.994\,\mathrm{mmol}$	$(13.8994, 1389.94)$	Yes

The indices match those in Fig 4. True values describe the parameters used to generate the data. Bounds are described as upper and lower bounds on parameters and define the parameter sampling region. If the parameters are log-transformable, then they are sampled from a log-uniform distribution rather than a uniform distribution. Log-transformable describes which parameters are transformed when log transforms are enabled. Units differ from [3] to ensure model consistency.

Table D. Loewe I_{Kur} parameters.

Index	True value	Bounds	Log-transformable
1	$0.65\,ms^{-1}$	$(0.065, 6.5)$	Yes
2	$10\,mV$	$(-50, 70)$	No
3	$8.5\,mV$	$(0.85, 85)$	Yes
4	$30\,mV$	$(-30, 90)$	No
5	$59\,mV$	$(5.9, 590)$	Yes
6	2.5	$(-57.5, 62.5)$	No
7	$82\,mV$	$(22, 142)$	No
8	$17\,mV$	$(1.7, 170)$	Yes
9	$30.3\,mV$	$(-29.7, 90.3)$	No
10	$9.6\,mV$	$(0.96, 960)$	Yes
11	3	$(0.3, 30)$	Yes
12	$1\,ms^{-1}$	$(0.1, 10)$	Yes
13	21	$(-39, 81)$	No
14	$185\,mV$	$(125, 245)$	No
15	$28\,mV$	$(2.8, 280)$	Yes
16	$158\,mV$	$(98, 218)$	No
17	$16\,mV$	$(1.6, 160)$	Yes*
18	$99.45\,mV$	$(39.45, 159.45)$	No
19	$27.48\,mV$	$(2.748, 274.8)$	Yes
20	3	$(0.3, 30)$	Yes
21	$0.005\,nS\,pF^{-1}$	$(-59.995, 60.005)$	No
22	$0.05\,nS\,pF^{-1}$	$(0.005, 0.5)$	Yes
23	$15\,mV$	$(-45, 75)$	No
24	$13\,mV$	$(1.3, 130)$	Yes
25	$138.994\,mmol$	$(13.8994, 1389.94)$	Yes

The indices match those in Fig 4. True values describe the parameters used to generate the data. Bounds are described as upper and lower bounds on parameters and define the parameter sampling region. If the parameters are log-transformable, then they are sampled from a log-uniform distribution rather than a uniform distribution. Log-transformable describes which parameters are transformed when log transforms are enabled. Units differ from [3] to ensure model consistency. * This parameter was originally labelled as additive [3], but its appearance in the model and the originally used parameter bounds suggest it should be treated as multiplicative, so we allow it to be log transformed.

Table E. Moreno I_{Na} parameters.

Index	True value	Bounds	Log-transformable
1	7.6178×10^{-3} ms	$(5.71 \times 10^{-3}, 9.52 \times 10^{-3})$	Yes
2	32.764 mV	$(24.6, 41)$	No
3	5.8871×10^{-1}	$(4.42 \times 10^{-1}, 7.36 \times 10^{-1})$	Yes
4	1.5422×10^{-1}	$(1.16 \times 10^{-1}, 1.93 \times 10^{-1})$	Yes
5	2.5898 ms	$(1.94, 3.24)$	Yes
6	8.5072 mV	$(6.38, 10.6)$	No
7	1.3760×10^{-3}	$(1.03 \times 10^{-3}, 1.72 \times 10^{-3})$	Yes
8	2.888	$(2.17, 3.61)$	Yes
9	3.2459×10^{-5} ms^{-1}	$(2.43 \times 10^{-5}, 4.06 \times 10^{-5})$	Yes
10	9.5951 mV	$(7.20, 12)$	No
11	1.3771 ms^{-1}	$(1.03, 1.72)$	Yes
12	21.126 mV	$(15.8, 26.4)$	No
13	11.086 ms^{-1}	$(8.31, 13.9)$	Yes
14	43.725 mV	$(32.8, 54.7)$	No
15	4.1476×10^{-2}	$(3.11 \times 10^{-2}, 5.18 \times 10^{-2})$	Yes
16	2.0802×10^{-2}	$(1.56 \times 10^{-2}, 2.60 \times 10^{-2})$	Yes

The indices match those in Fig 4. True values describe the parameters used to generate the data. Bounds are described as upper and lower bounds on parameters (rounded to 3 significant figures here) and define the parameter sampling region. Log-transformable describes which parameters are transformed when log transforms are enabled.

A.5 Solver tolerances

The ODE solver tolerances for each problem are given in Table F. The script used to verify the accuracy of the solver at these tolerances, and its output as a text file, are given in the GitHub repository.

The solver tolerances are set such that three conditions are satisfied. The first two conditions concern the magnitude of the solver noise (calculated as the standard deviation of the cost around a parameter vector) across the parameter space, and the third concerns the cost threshold.

The first condition samples parameters across the whole parameter space, calculates the local standard deviation in the cost for small ($\mathcal{O}(10^{-12})$) perturbations in parameters, and verifies the median is below 10^{-7}. The second condition is that the 75th percentile of the standard deviation of the cost is below 10^{-6}. The third condition verifies that points near to the true parameters always satisfy the cost threshold for the problem.

Note that only the Staircase problems use the ODE solver for the cost function evaluation. The other problems use an analytical solver, and use the specified tolerances only for solves with sensitivities, as analytical solves with sensitivities are not available in `myokit`.

Table F. ODE solver tolerances.

Problem	Abs. tolerance	Rel. tolerance
Staircase HH	10^{-5}	10^{-5}
Staircase MM	10^{-6}	10^{-6}
Loewe I_{Kr}	10^{-6}	10^{-6}
Loewe I_{Kur}	10^{-8}	10^{-8}
Moreno I_{Na}	10^{-7}	10^{-7}

The absolute and relative solver tolerances used for each problem.

B Model solve time

One of the assumptions in the results is that the model always takes the same amount of time to solve, regardless of the approach. It is possible that some approaches or optimisers propose more or less stiff parameters which may lead to a corresponding change in the time to solve the model for those parameters.

In addition to recording the number of function evaluations in `ionBench`, we also record the time to solve the model. These are reported in Figs A and B for the solves without and with sensitivities, respectively. It is clear that the average solve time for the models is independent of the choice of approach, meaning it is reasonable to use function evaluations in place of model solve time for the ERT calculations.

There is an outlier in the Moreno I_{Na} problem cost timings in Fig A. This point corresponds to the Sachse2003b approach. During all the optimisations with this approach, the first two points were correctly solved, the third point resulted in a failed solved (giving a NaN cost), and the remaining solves were all attempted at NaN parameters. All optimisations resulted in 48 model solves (all without sensitivities), but because these solves failed, they were able to fail quickly. Since the average model solve times for each problem are weighted by the number of solves for each approach, this outlier has not affected the average model solve time for the Moreno I_{Na} problem significantly.

C Significance

C.1 Bootstrapping algorithm

In this section, we describe the bootstrapping algorithm used to verify the significance of the results in `ionBench`.

Algorithm A describes how a single bootstrapped ERT sample is generated. Algorithm B describes how we compare the ERT samples for the best approach against each other approach.

Algorithm C demonstrates how we use this to run all approaches until they achieve significant results.

C.2 Significance

All approach-problem pairs that were not significantly worse than the Wilhelms2012b approach paired with that problem were rerun with an unbounded number of maximum runs. They (both the approach to be evaluated and Wilhelms2012b) were run against 50 parameter samples (10 for the Staircase HH problem) and the significance was evaluated. If the Wilhelms2012b approach was not found to be significantly better or significantly worse, then a further 50 (or 10) parameters were sampled and evaluated against. This continued until the Wilhelms2012b approach was significantly better or worse than each other approach-problem pairing.

Table G presents the number of runs required for each approach before the results became significant. In all cases, except for the Dokos2004-Staircase HH pairing, the results are significant in favour of the Wilhelms2012b approach.

The parameters used for the significance testing for a given problem are consistent across approaches, but are different from the initial n_{Run} parameters.

There are also a couple of edge cases, where slightly different methods were used to improve computation times. In one case (Chen2012 approach for the Staircase MM problem), the batch size was unsuitable due to long computational times, so a smaller batch size of 5 was used. While generally, the approaches were tested against the same parameters as Wilhelms2012b, for the Groenendaal2015 approach (and Staircase MM problem), 2500 parameters were used for Wilhelms2012b (to reduce its uncertainty) and Groenendaal2015 was then run in batches (of size 10) until significance. Otherwise, Groenendaal2015 likely would not have seen significance until around 400 evaluated parameters. Similarly, 5000 parameters were used for Wilhelms2012b and Bueno-Orovio2008 was allowed to continue until significance (in this case, over 5000 parameters).

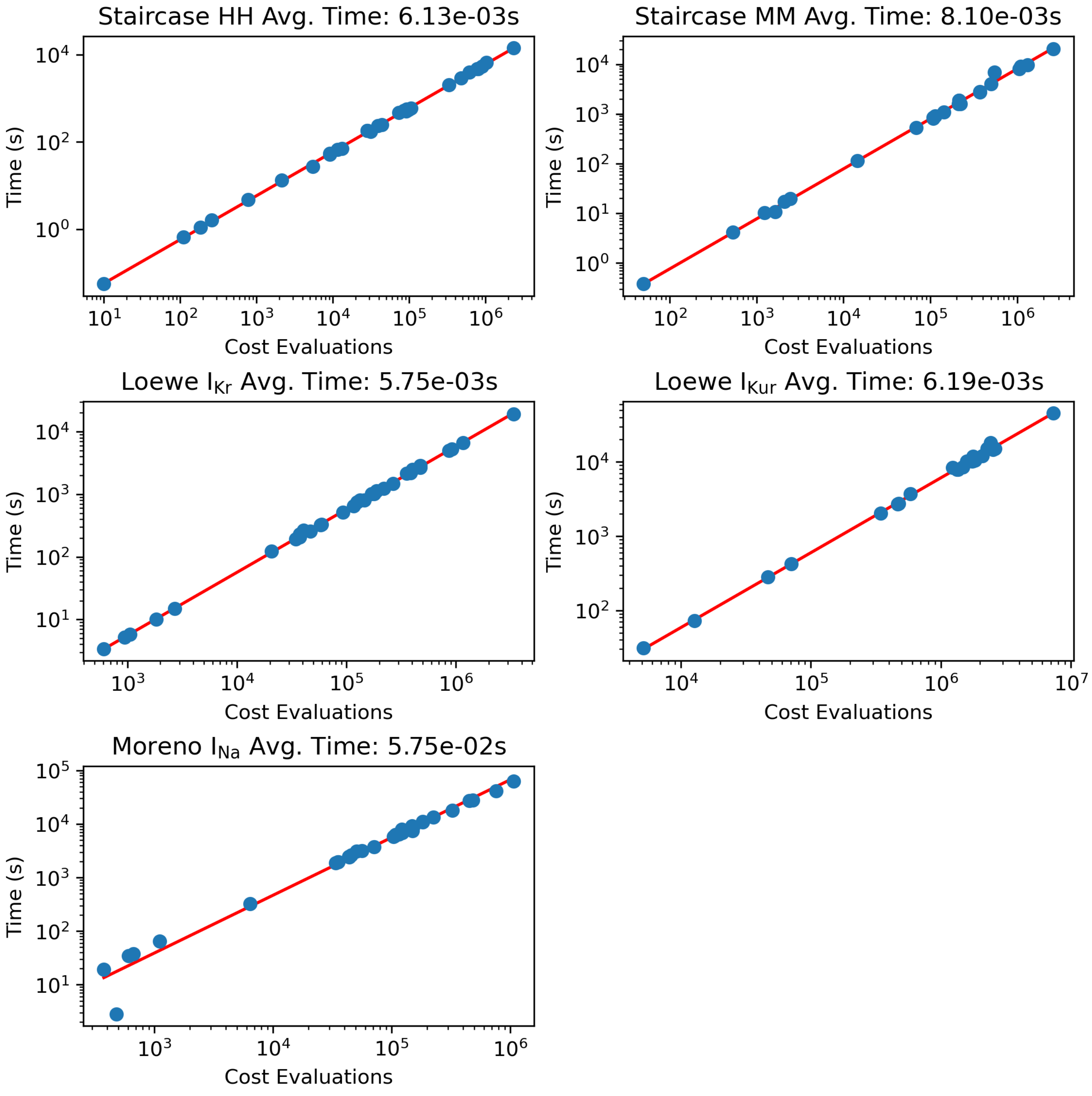

Fig A. Time to evaluate cost. Plots of the total time spent solving the model (without sensitivities) against the number of function evaluations for each approach (cumulative across the n_{Runs}).

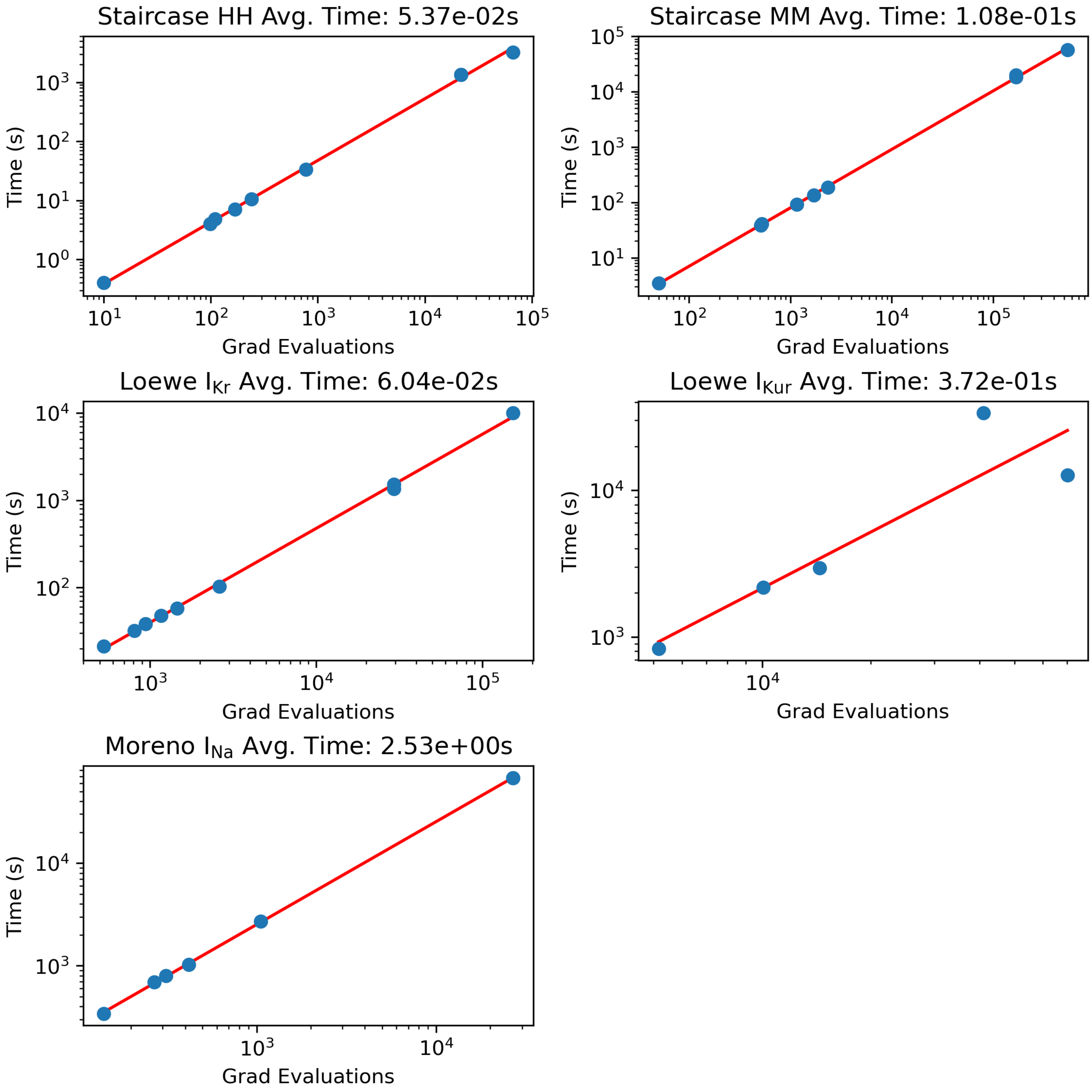

Fig B. Time to evaluate gradient. Plots of the total time spent solving the model (with sensitivities) against the number of function evaluations for each approach (cumulative across the n_{Runs}).

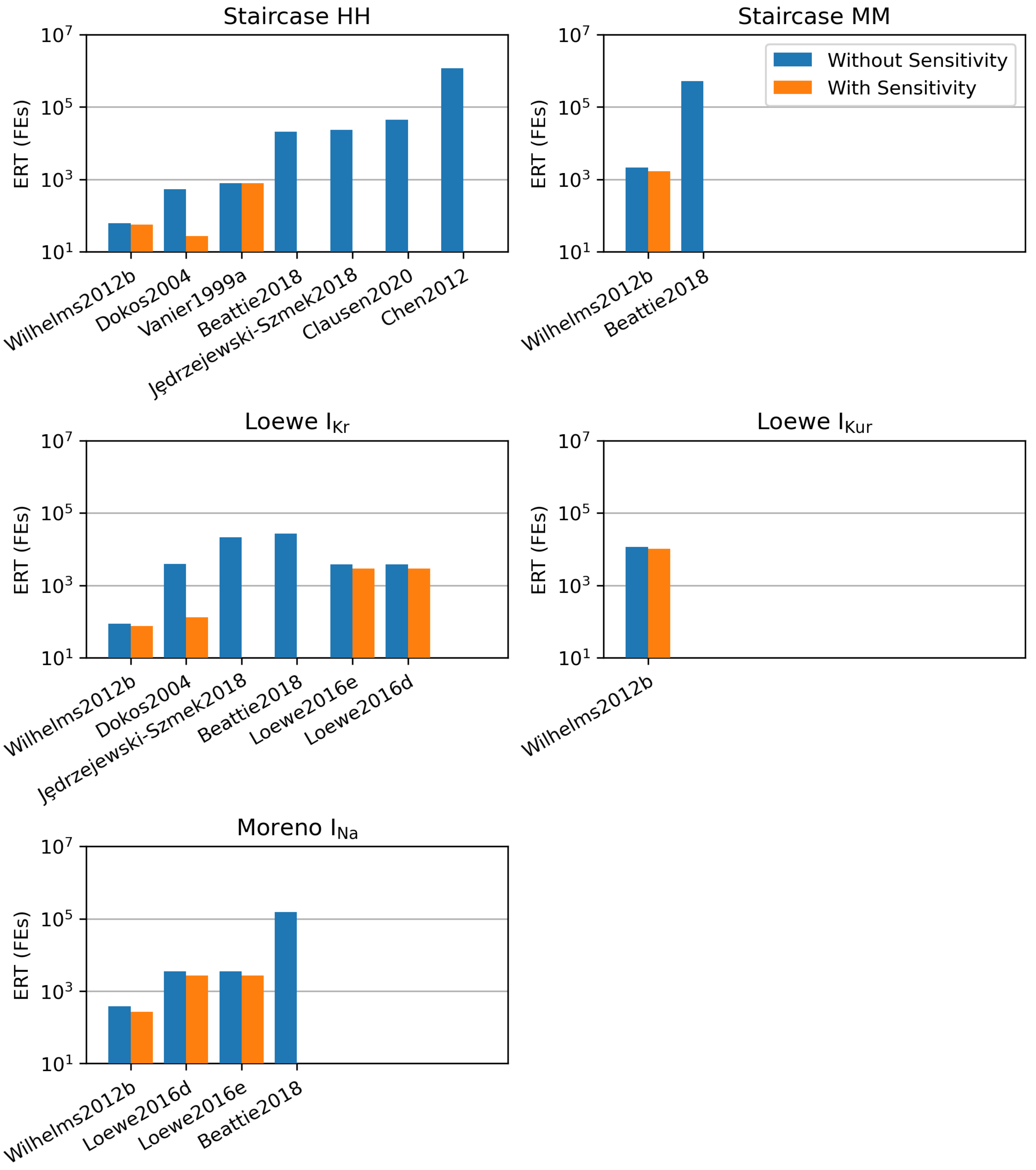

Fig C. ERT of successful approaches, split into cost and gradient. Plots of ERT, separated into solves with and without sensitivities. The total ERT, as shown in Fig 6, is given as the without sensitivity ERT plus the with sensitivity ERT after being scaled by the time ratio in Table 3.

Algorithm A: Draw a single sample of ERT using data from a particular approach and problem

```
Input: t: List of times for an approach. s: List of success/failures for an approach
Output: ERT: Sampled ERT for bootstrapping
nRuns = len(t);
Draw bootstrap sample of optimisation runs
mask = choose(from = 1 : nRuns, count = nRuns, replacement = True);
s_bs = s[mask];
t_bs = t[mask];
Sample bootstrapped success rate
p ~ Beta(α = 0.5 + sum(s_bs == True), β = 0.5 + sum(s_bs == False));
p_bs = drawSample(p);
Calculate average success and fail times
if sum(s_bs == success) > 0 then
  | T_s = mean(t_bs[s_bs == success]);
else
  | Choosing a single value maximises the bootstrapped variances when we are
  |  most unsure about T_s
  | T_s = choose(from = t_bs, count = 1);
end
if sum(s_bs == fail) > 0 then
  | T_f = mean(t_bs[s_bs == fail]);
else
  | Choosing a single value maximises the bootstrapped variances when we are
  |  most unsure about T_f
  | T_f = choose(from = t_bs, count = 1);
end
ERT = T_s + T_f × (1 − p_bs)/p_bs;
```

Algorithm B: The bootstrapping algorithm for comparing against the best approach.

```
Input: B: number of bootstrapped samples per approach; T[i]: list of run times for
       approach i; S[i]: list of boolean success/failures for approach i.
Output: sig: List of length n = len(T[i]) = len(S[i]) boolean indicating if approach 1 is
        significantly better than approach i.
Sort T and S by ERT such that T[1] and S[1] correspond to the approach that gives the
 lowest ERT;
Get number of approaches
n = len(T);
Generate bootstrapped samples for each approach
for i in 1 to n do
    Sample B ERTs for approach i
    for b in 1 to B do
        ERT[i][b] = Algorithm A(T[i], S[i]));
    end
end
Compare approaches for significance
for i in 1 to n do
    if i==1 then
        No point in comparing approach 1 against itself
        sig[i] = N/A;
    else
        count = 0;
        for b_1, b_2 in 1 to B do
            if ERT[i][b_1] < ERT[1][b_2] then
                count = count + 1;
            end
        end
        count = count/B^2;
        if count > 0.05 then
            Approach i is better in over 5% of samples
            sig[i] = False;
        else
            Approach 1 is better in over 95% of samples
            sig[i] = True;
        end
    end
end
```

Algorithm C: Performing longer runs to verify significance.

Input: Test approach to compare against Wilhelms2012b.

Output: n_{Run}.

Sort T and S by ERT such that T[1] and S[1] correspond to the approach that gives the lowest ERT;

$n_{\text{Run}} = 0$;

$data_{\text{test}} = []$;

$data_{\text{Wilhelms2012b}} = []$;

while *not Significant* **do**

    `Run test approach for 100 new parameters`

    `Append new data to` $data_{\text{test}}$

    `Run Wilhelms2012b on same parameters`

    `Append new data to` $data_{\text{Wilhelms2012b}}$

    Significant <- Algorithm B($data_{\text{test}}$, $data_{\text{Wilhelms2012b}}$);

    $n_{\text{Run}} += 100$;

end

`Report which of test or Wilhelms2012b were significantly better than the other`

Table G. Number of runs required for significance.

Approach	Staircase HH	Staircase MM	Loewe I_{Kur}	Moreno I_{Na}
Balser1990a	40	1200	N/A	50
Balser1990b	A/S	400	A/S	50
Vanier1999a	A/S	600	750	50
Vanier1999b	A/S	400	A/S	50
Vanier1999c	A/S	3750	300	50
Vanier1999d	A/S	400	350	50
Sachse2003a	A/S	600	A/S	N/A
Sachse2003b	A/S	400	450	350
Dokos2004	80	2350	A/S	150
Bueno-Orovio2008	160	7950	450	50
Seemann2009a	A/S	400	A/S	A/S
Liu2011	A/S	400	A/S	A/S
Chen2012	A/S	N/A	N/A	45
Davies2012	A/S	400	A/S	50
Groenendaal2015	A/S	20	A/S	A/S
Loewe2016d	A/S	A/S	N/A	100
Loewe2016e	A/S	A/S	N/A	100
Moreno2016	A/S	400	A/S	50
Beattie2018	A/S	600	A/S	50

The number of runs required to show significance. Duplicate approaches (see Table 1) are not included since their results are identical to a reported approach. The remaining approaches that are not shown were already significantly worse for all problems. The Loewe I_{Kr} problem is not shown since Wilhelms2012b was significantly better than all other approaches. A/S means that the Wilhelms2012b approach was already significantly better and further runs were not needed. N/A means the approach-problem paring failed to complete the initial n_{Run} optimisations in the 1 week limit, or otherwise failed (e.g. the Carins2017 approach is incompatible with negative parameters). Cells highlight in blue show where an approach was significantly better than Wilhelms2012b, otherwise the Wilhelms2012b approach was significantly better.

D Profile likelihood

Profile likelihood plots for the problems are given in Figs D, E, F, G, and H. Since not all of the problems have noise, we report the RMSE cost rather than the likelihood (negative log likelihood is linearly related to the square of the cost), though still referred to here as profile likelihood curves.

These figures are generated through ionBench and use the TRR optimiser. The code to generate the profile likelihood plots is given in the GitHub repository.

We report both 'optimised' curves where the cost is optimised, keeping only one parameter fixed, and 'unoptimised' curves where all parameters keep their default values except for the parameter fixed by the x-axis. The unoptimised curves give a sense of scale of the cost function surface by providing a linear slice through it (if both curves are the same and flat, then the parameter can be perturbed with minimal change to the cost, while if they are very different, then changes in the parameter can be accommodated by changes in other parameters).

The greater the curvature of the profile likelihood curve around the data generating parameters, the better the identifiability of that parameter. Parameters which are unidentifiable will have little or no discernable curvature (aside from ODE solver noise). If the minimum of the profile likelihood curve is not at $x = 1$ (with $x = 1$ representing the default value of the parameters), then the global minimum is not at the data generating parameters. In our case, this can only be due to the inclusion of noise in the Staircase problems.

Our goal with the profile likelihood curves is to identify a reasonable cost threshold (rather than typically where the goal is to investigate individual parameters for unidentifiability). This cost threshold should be sufficient (at least approximately) that if a cost below the cost threshold is identified, then it implies all (identifiable) parameters are within 5% of the global data generating parameters or the global minimum. To identify which parameters we wish to include in the calculation of the cost threshold, we need to remove parameters with flat profile likelihood plots (both flat at zero and non-zero values) and parameters which see a large perturbation in the global minimum.

Since the Staircase MM shows very flat profile likelihood curves from some parameters, a figure where each plot is placed on its own scale is given in Fig I. This clearly highlights a shift in the global minimum. This shift is the result of the noise added to the data, where the model can attempt to reproduce some small bias by moving to parameters offset from those that generated the data. This has been previously reported [12], albeit with a smaller bias in parameters (likely due to using a stiffer model/parameterisation here). We have verified resampling the noise moves the bias in the minimum, with the distribution centred on the data-generating parameters, and shrinking the noise reduces the bias in the parameters (although too slowly to allow both noise and minimal bias in the parameter minimum).

The cost thresholds are calculated from the profile likelihood curves of each problem. We begin by identifying the cost of the profile likelihood curves at $\pm 5\%$ around the true parameters (data-generating). We then look at taking the minimum of these values, across all profile likelihood curves for a given problem, with some exceptions. We want to ignore any unidentifiable parameters, and also ensure the cost threshold is far enough away from the minimum that approaches that implement a function tolerance termination criteria do not abort early (which would require editing hyperparameters to resolve).

The unidentifiable parameters that are removed are parameters 1, 2, 4, 6, 7, 11, 12, 13, 14, 16, and 20 of the Loewe I_{Kur} problem (implemented as ignoring parameters if their perturbed cost is below 10^{-14}). The limit of 10^{-14} is specific to the profile likelihood curves here and will not work in general for other unknown problems. This removes any parameters which have a flat profile likelihood where the curve is flat at (or rather near to) zero.

We also need to ignore any parameters where the perturbed cost (at the $\pm 5\%$ positions) is below the cost of the data-generating parameters. This is relevant for the Staircase MM problem, which sees a significant noise-induced bias in the global minimum. If this step was not done, then the cost threshold could end up arbitrarily close to the global minimum, depending on the cost of the perturbed parameters. This removes any parameters where we see a large perturbation in the global minimum.

Next, we find the minimum of the perturbed costs, across all profile likelihood curves for each problem, ignoring any points that meet the above criteria.

Finally, we want to ensure there is some buffer room in the cost threshold. To do this, we will round the cost threshold up to 3 significant figures. If this step was not taken, then some problems, particularly Staircase MM where the cost function is very flat, would trigger many different termination criteria (function and gradient tolerances) too early, and these approaches would never be able to identify a minimum without changing hyperparameters, even if they reproduce the data almost perfectly. This effectively removes the parameters where the profile likelihood is flat at non-zero values.

The Loewe I_{Kur} problem sees many unidentifiable parameters. Of the 25 profile likelihood curves in Fig G, 11 show unidentifiability up to optimisation tolerances (parameters 1, 2, 4, 6, 7, 11, 12, 13, 14, 16, and 20).

The Moreno I_{Na} problem, shown in Fig H, shows some optimisation difficulties in generating the profile likelihood plots. In all cases, either the profile likelihood curves at the ±5% points that are used to determine the cost thresholds appear to be accurate, or a small portion of the smooth profile likelihood curve can be seen and is well above the cost threshold. This suggests that these difficulties (which we believe are due to the post processing to calculate the summary statistics) are unlikely to influence the cost threshold calculation.

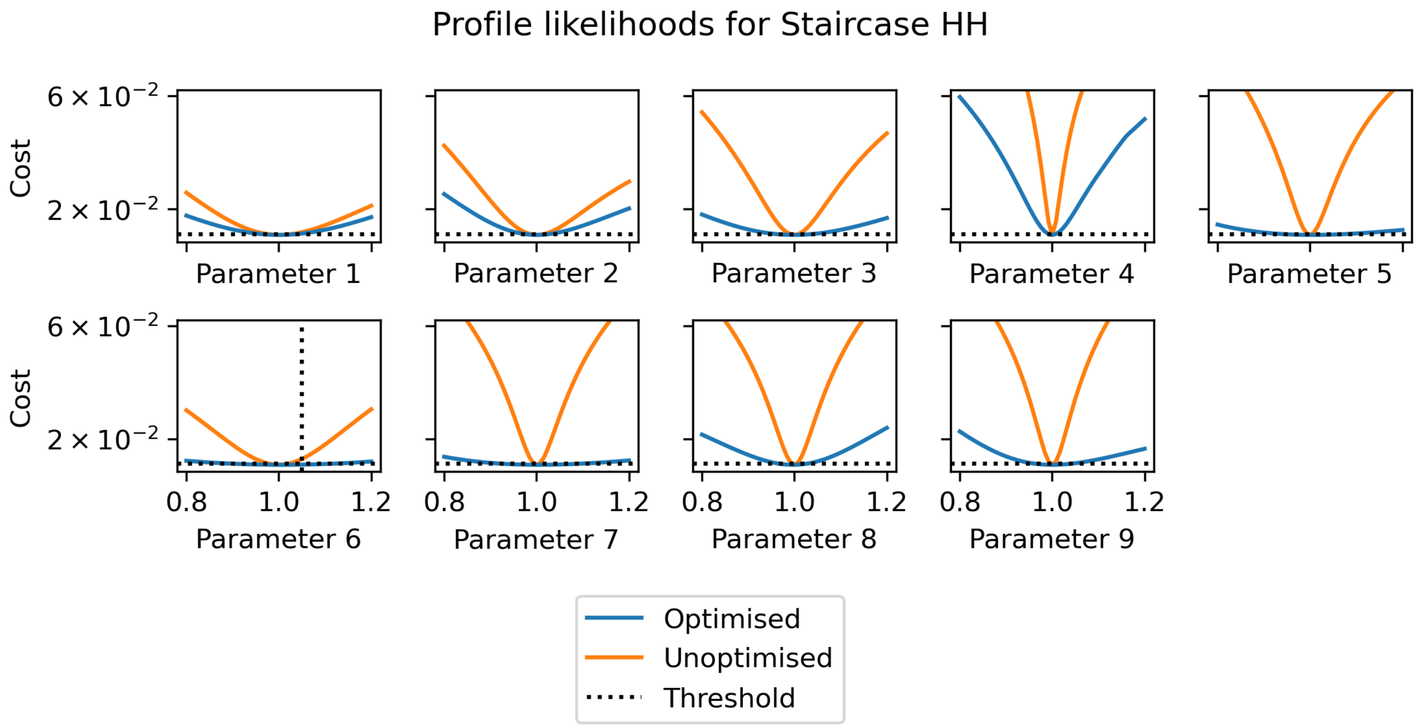

Fig D. Profile likelihood plots of the Staircase HH problem. The RMSE cost is given rather than likelihood, so the maximum likelihood estimates are given at the minimum cost. The true parameters are at $x = 1$. Both the profile likelihood curve (blue) and an unoptimised cost surface slice (orange) are shown. The cost threshold is shown as a horizontal dotted line, and the point which determines its value is labelled with a vertical dotted line.

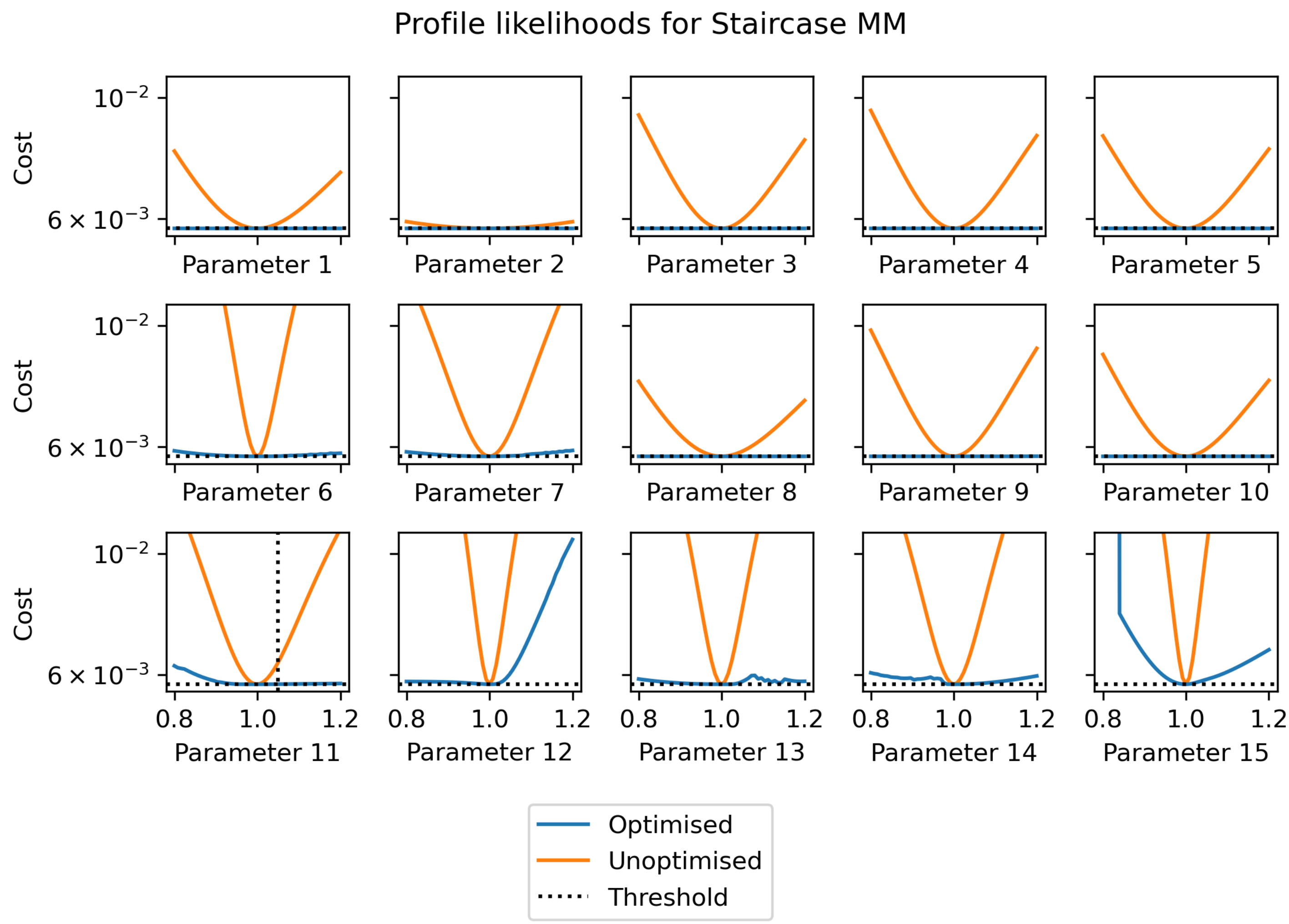

Fig E. Profile likelihood plots of the Staircase MM problem. The RMSE cost is given rather than likelihood, so the maximum likelihood estimates are given at the minimum cost. The true parameters are at $x = 1$. Both the profile likelihood curve (blue) and an unoptimised cost surface slice (orange) are shown. The cost threshold is shown as a horizontal dotted line, and the point which determines its value is labelled with a vertical dotted line.

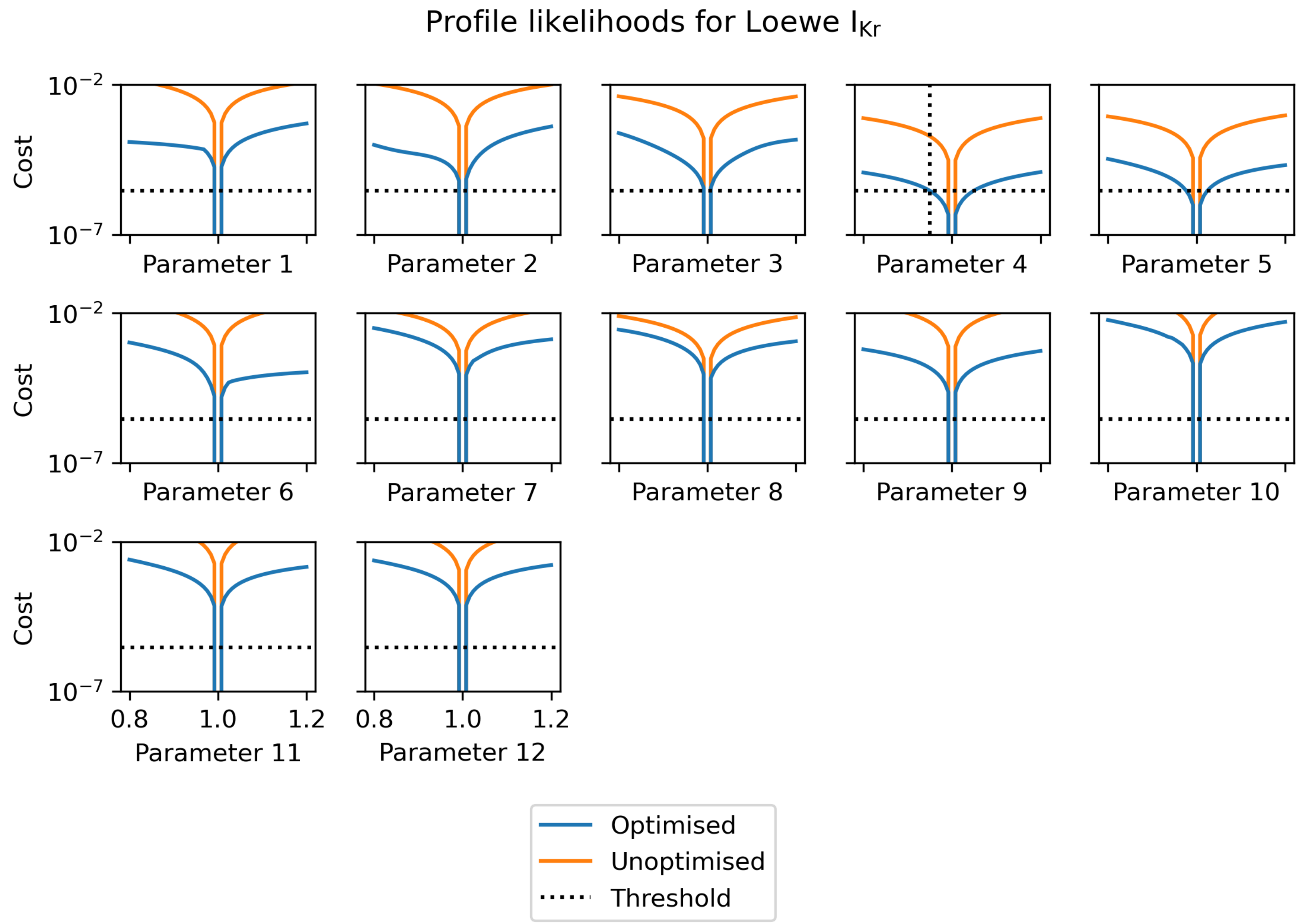

Fig F. Profile likelihood plots of the Loewe $\mathbf{I_{Kr}}$ problem. The RMSE cost is given rather than likelihood, so the maximum likelihood estimates are given at the minimum cost. The true parameters are at $x = 1$. Both the profile likelihood curve (blue) and an unoptimised cost surface slice (orange) are shown. The cost threshold is shown as a horizontal dotted line, and the point which determines its value is labelled with a vertical dotted line.

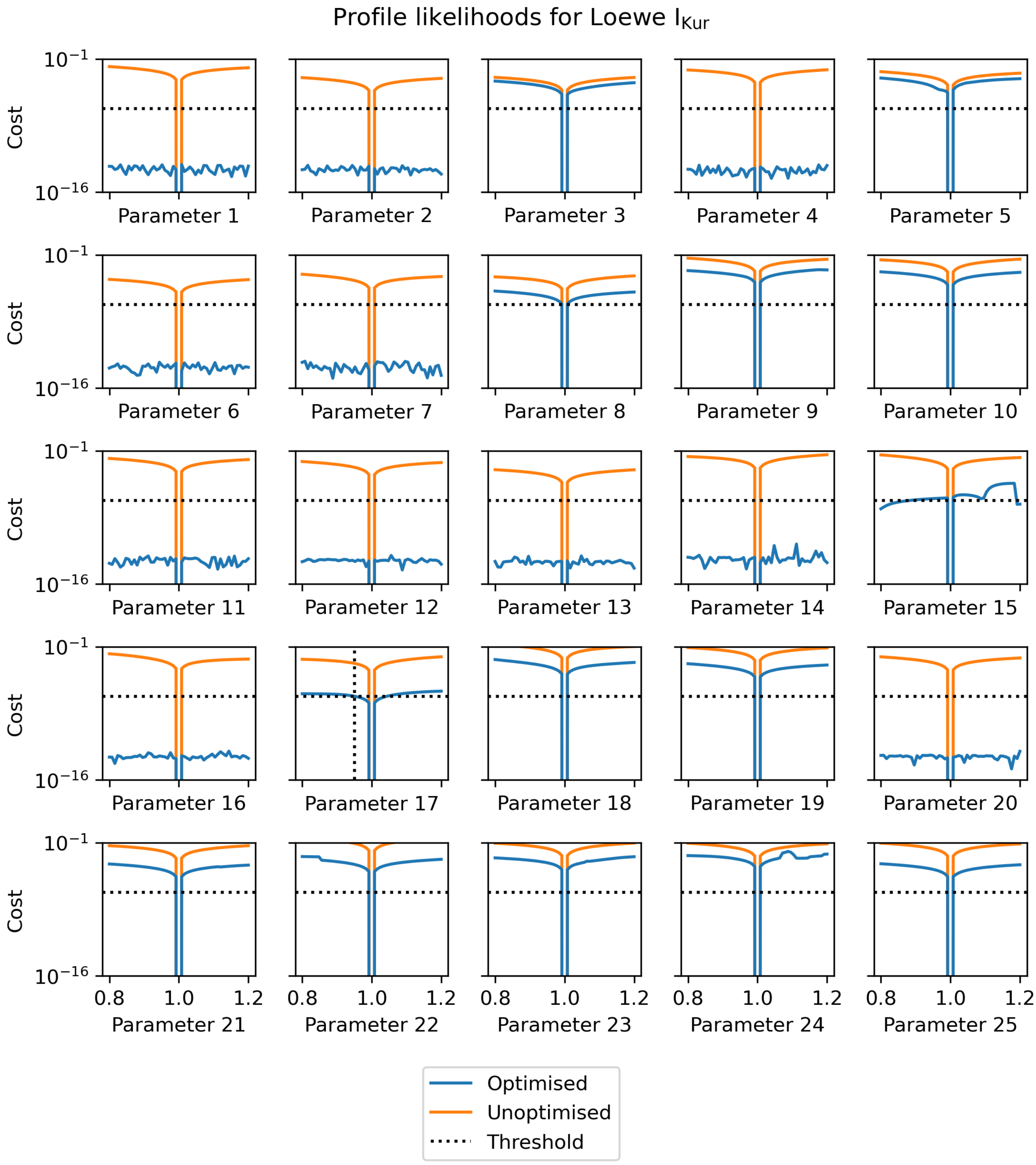

Fig G. Profile likelihood plots of the Loewe I_{Kur} problem. The RMSE cost is given rather than likelihood, so the maximum likelihood estimates are given at the minimum cost. The true parameters are at $x = 1$. Both the profile likelihood curve (blue) and an unoptimised cost surface slice (orange) are shown. The cost threshold is shown as a horizontal dotted line, and the point which determines its value is labelled with a vertical dotted line.

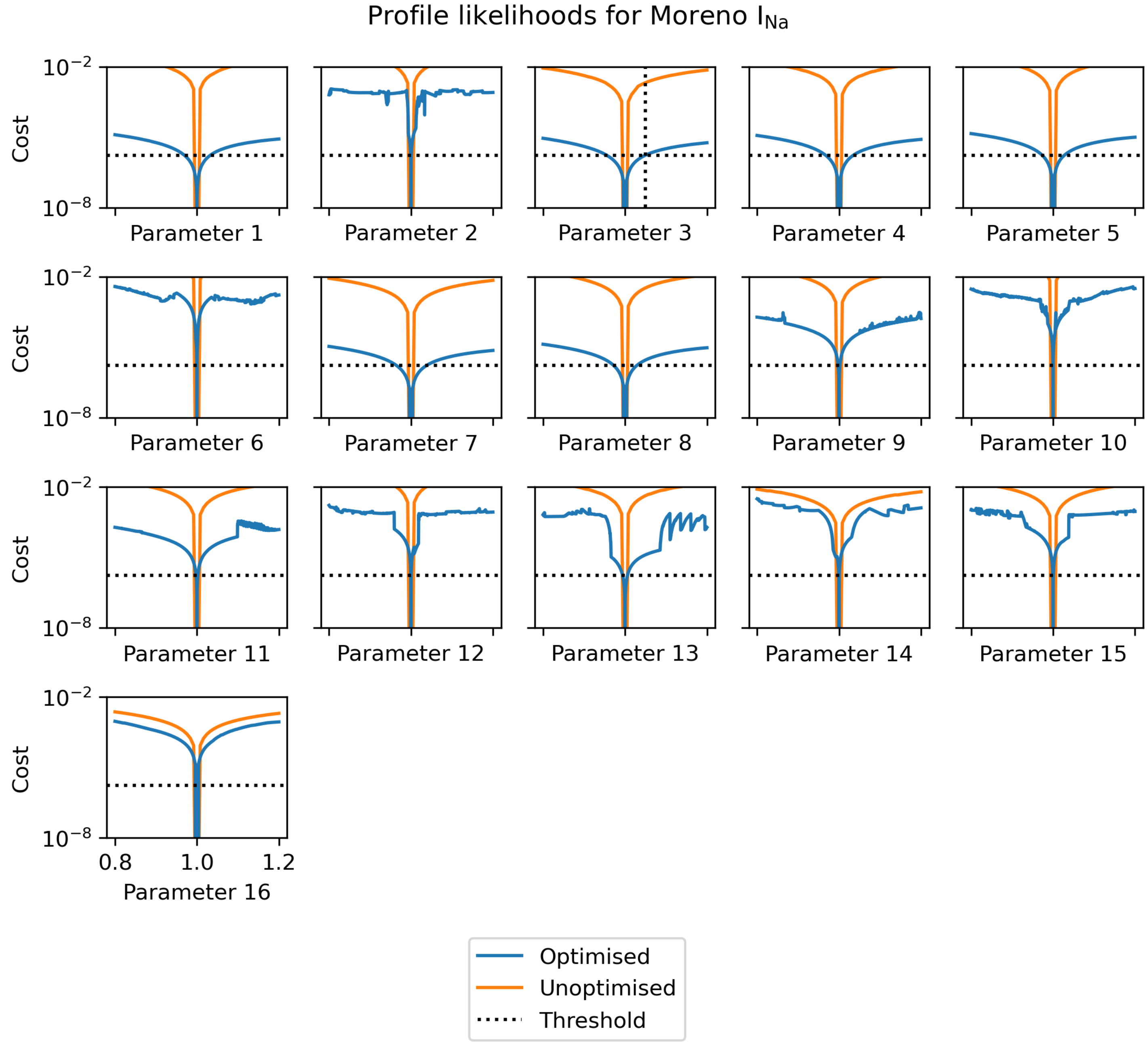

Fig H. Profile likelihood plots of the Moreno I_{Na} problem. The RMSE cost is given rather than likelihood, so the maximum likelihood estimates are given at the minimum cost. The true parameters are at $x = 1$. Both the profile likelihood curve (blue) and an unoptimised cost surface slice (orange) are shown. The cost threshold is shown as a horizontal dotted line, and the point which determines its value is labelled with a vertical dotted line.

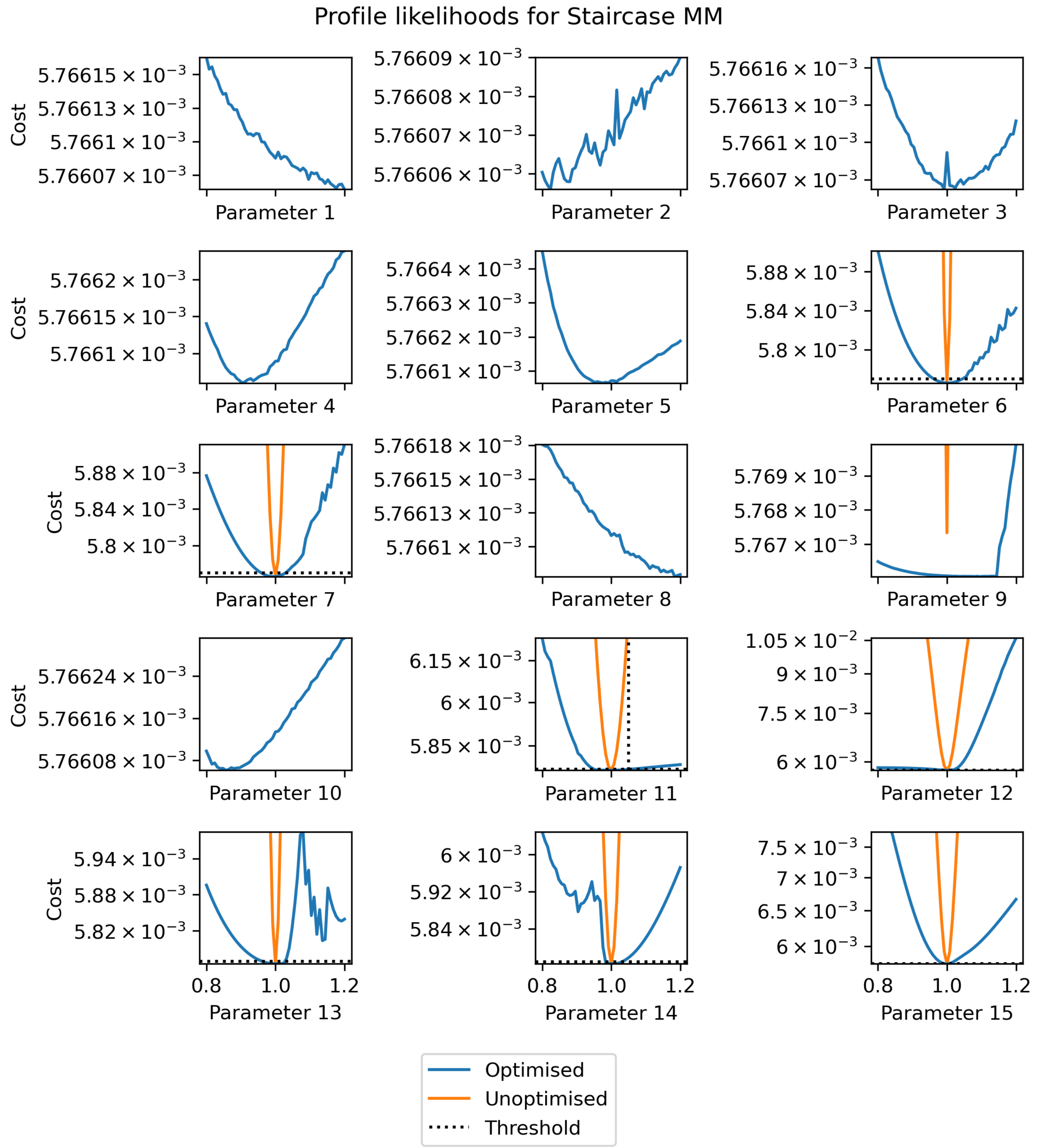

Fig I. Duplicate of Fig E, but each subplot is on an independent scale. Profile likelihood plots of the Staircase MM problem. The RMSE cost is given rather than likelihood, so the maximum likelihood estimates are given at the minimum cost. The true parameters are at $x = 1$. Both the profile likelihood curve (blue) and an unoptimised cost surface slice (orange) are shown. The cost threshold is shown as a horizontal dotted line, and the point which determines its value is labelled with a vertical dotted line.

E Performance of all approaches

Reproductions of Fig 7, isolated to the Staircase HH, Staircase MM, Loewe I_{Kr}, Loewe I_{Kur}, and Moreno I_{Na} problems, are given in Figs J, K, L, M, and N, respectively. The raw data, cost and time of the best run for each approach-problem paring, from Fig 7 is given in Table H.

Table H. Best run for each approach-problem pairing.

Name	Staircase HH		Staircase MM		Loewe I_{Kr}		Loewe I_{Kur}		Moreno I_{Na}	
	Cost	Time	Cost	Time	Cost	Time	Cost	Time	Cost	Time
Balser1990a	2.44e-1	2.02e2	5.84e-2	1.91e3	1.94e-3	3.03e3	7.46e-2	1.40e4	1.21e-3	4.80e3
Balser1990b	1.84e-1	1.81e3	3.05e-2	1.37e4	5.95e-2	3.39e3	1.66e-2	2.00e4	9.48e-4	6.60e3
Maryak1998	2.83e-1	3.21e3	NA	NA	3.31e-3	7.13e4	NA	NA	2.41e-3	2.13e4
Vanier1999a	1.57e-2	4.74e3	1.06e-1	1.43e1	2.30e-6	1.88e4	4.39e-2	1.65e4	1.15e-6	4.39e4
Vanier1999b	1.22e-1	3.36e3	2.59e-2	1.16e4	4.50e-3	1.66e4	3.12e-2	1.37e4	3.68e-3	6.60e3
Vanier1999c	3.24e-1	5.26e2	4.55e-2	3.42e3	3.03e-3	3.06e3	4.07e-2	2.06e4	3.90e-3	3.41e3
Vanier1999d	1.78e-1	4.17e3	4.42e-2	9.82e3	1.71e-2	1.23e4	3.79e-2	7.11e4	3.21e-3	1.71e4
Sachse2003a	3.56e-1	2.05e2	4.55e-2	3.42e3	9.24e-2	1.60e4	2.78e-2	1.78e4	6.77e-4	4.26e3
Sachse2003b	2.13e-2	1.63e4	2.69e-2	8.45e3	3.21e-4	1.08e4	4.50e-2	4.18e4	7.19e-4	1.27e4
Dokos2004	1.56e-2	7.58e2	5.77e-3	1.93e4	1.98e-6	3.45e3	2.16	1.54e3	1.15e-6	4.39e4
Gurkiewicz2007a	3.36e-2	8.53e4	NA	NA	1.32e-3	1.20e5	NA	NA	6.15e-4	1.28e5
Gurkiewicz2007b	1.59e-2	4.78e4	NA	NA	1.24e-1	3.31e4	NA	NA	1.13e-3	5.86e4
Bueno-Orovio2008	3.86e-1	9.77	1.68e-2	1.56e4	2.20e-3	3.82e3	6.49e-2	1.31e5	1.00e-3	8.53e2
Seemann2009a	2.68e-1	4.74e3	5.93e-3	1.20e4	1.47e-4	2.00e4	2.37e-2	3.65e4	3.55e-3	2.34e4
Zhou2009	4.80e-2	8.54e4	8.67e-2	5.85e3	4.28e-2	2.91e4	NA	NA	6.32e-4	2.13e4
Liu2011	1.29e-1	1.18e3	6.88e-2	4.45e3	4.84e-3	6.85e3	1.37e-2	2.00e4	1.47e-3	5.96e3
Chen2012	1.57e-2	2.43e5	7.18e-3	3.91e3	3.71e-3	2.32e3	7.74e-7	6.44e3	7.64e-4	1.01e3
Davies2012	1.84e-1	2.17e3	6.06e-2	7.45e3	7.83e-2	1.08e4	3.95e-2	1.97e4	1.18e-3	4.90e3
Bot2012	4.35e-2	5.90e4	NA	NA	5.02e-3	1.39e5	NA	NA	1.43e-3	5.12e4
Ben-Shalom2012	2.65e-2	6.36e4	NA	NA	5.61e-3	1.77e5	NA	NA	3.23e-4	6.98e4
Wilhelms2012b	1.56e-2	1.91e2	5.77e-3	3.05e3	1.44e-6	8.63e2	2.31e-7	1.28e4	1.52e-6	6.17e2
Groenendaal2015	1.31e-1	1.54e4	1.04e-2	2.83e4	2.48e-3	2.59e4	1.28e-5	1.30e5	1.85e-3	1.59e4
Loewe2016b	NA	NA	NA	NA	1.44e-3	3.26e5	NA	NA	NA	NA
Loewe2016c	NA	NA	NA	NA	NA	NA	NA	NA	NA	NA
Loewe2016d	3.12e-1	1.75e4	1.04e-2	2.83e4	4.06e-3	4.91e4	NA	NA	1.27e-3	7.91e3
Loewe2016e	3.12e-1	1.75e4	6.82e-3	1.25e5	1.30e-2	2.10e4	8.14e-6	8.87e4	4.20e-3	1.65e4
Moreno2016	1.84e-1	1.81e3	5.66e-2	1.93e3	2.93e-3	7.22e3	2.69e-2	1.62e4	9.04e-3	3.45e3
Cairns2017	2.61e-1	3.05e3	1.60e-2	3.36e4	1.72e-2	2.03e4	3.15e-2	1.71e4	1.53e-3	1.43e4
Jędrzejewski-Szmek2018	1.57e-2	1.29e4	8.63e-3	7.50e2	2.50e-7	2.91e4	2.42e-1	4.42e3	4.44e-2	4.80e1
Beattie2018	1.57e-2	8.60e3	1.06e-1	1.50e1	2.56e-6	1.56e4	1.36e-2	5.64e3	1.66e-6	1.79e4
Smirnov2020	1.61e-2	2.61e4	NA	NA	3.31e-3	7.13e4	NA	NA	1.42e-3	1.77e4
Clausen2020	1.57e-2	7.32e3	8.22e-2	3.59e2	2.50e-7	2.91e4	2.08e-1	6.06e3	5.46e-4	8.32e2
Cabo2022	2.02e-1	1.12e4	5.78e-3	3.91e4	5.18e-2	1.26e4	7.29e-2	4.73e4	9.18e-3	1.80e4
Kohjitani2022	1.57e-2	8.34e4	NA	NA	5.59e-3	5.29e4	NA	NA	6.32e-4	2.13e4

The numeric data for Fig 7 showing the cost and time (in FEs) of the best run (lowest cost) for each approach-problem paring.

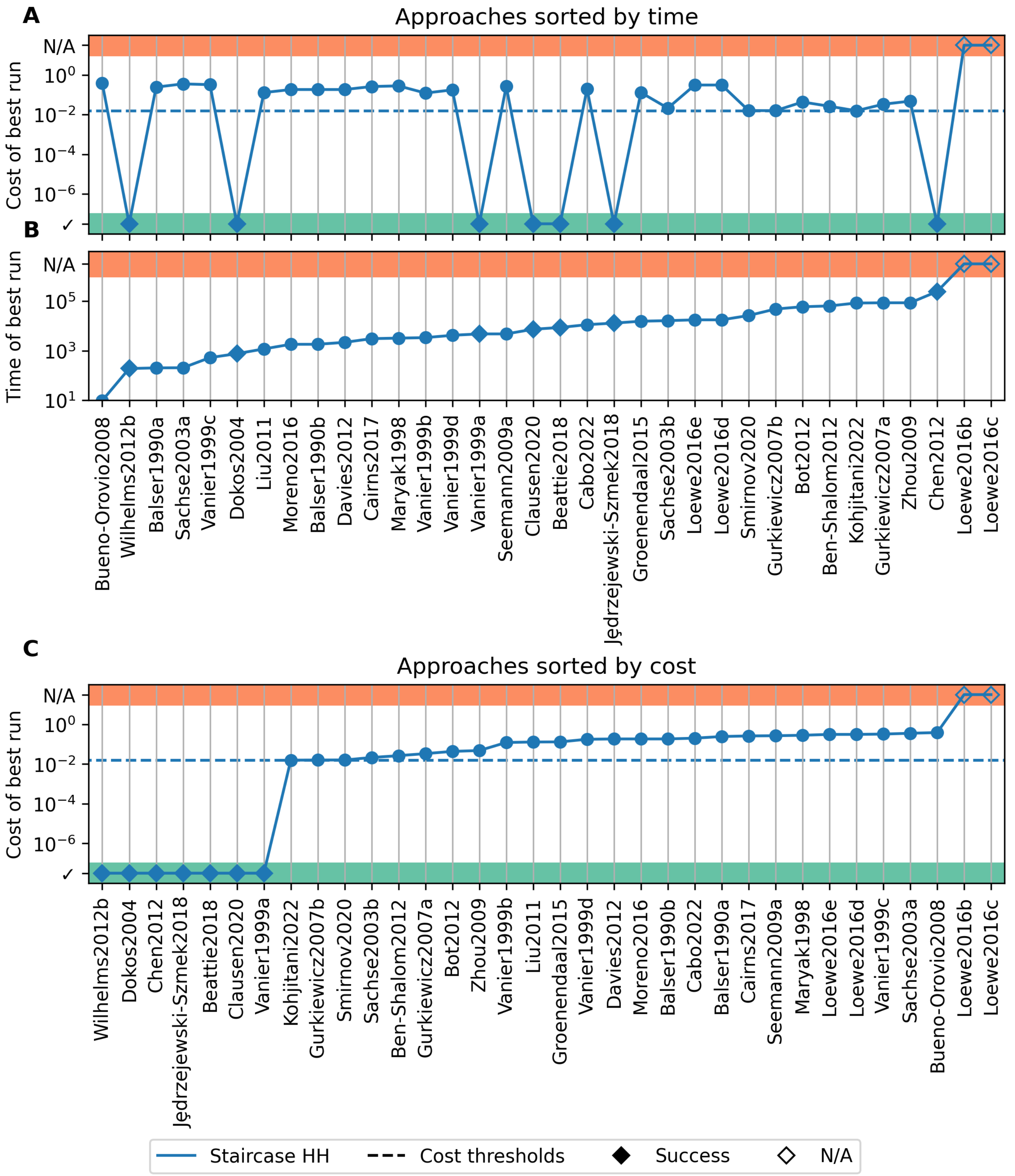

Fig J. Performance on Staircase HH. Cost or time (in FEs) of the best run for each approach on the Staircase HH problem, against approaches sorted by cost or time. (A) Cost, sorted by time. (B) Time, sorted by time. (C) Cost, sorted by cost. Cost thresholds are given as dashed lines. Successful runs are denoted by ✓; and incomplete runs by N/A.

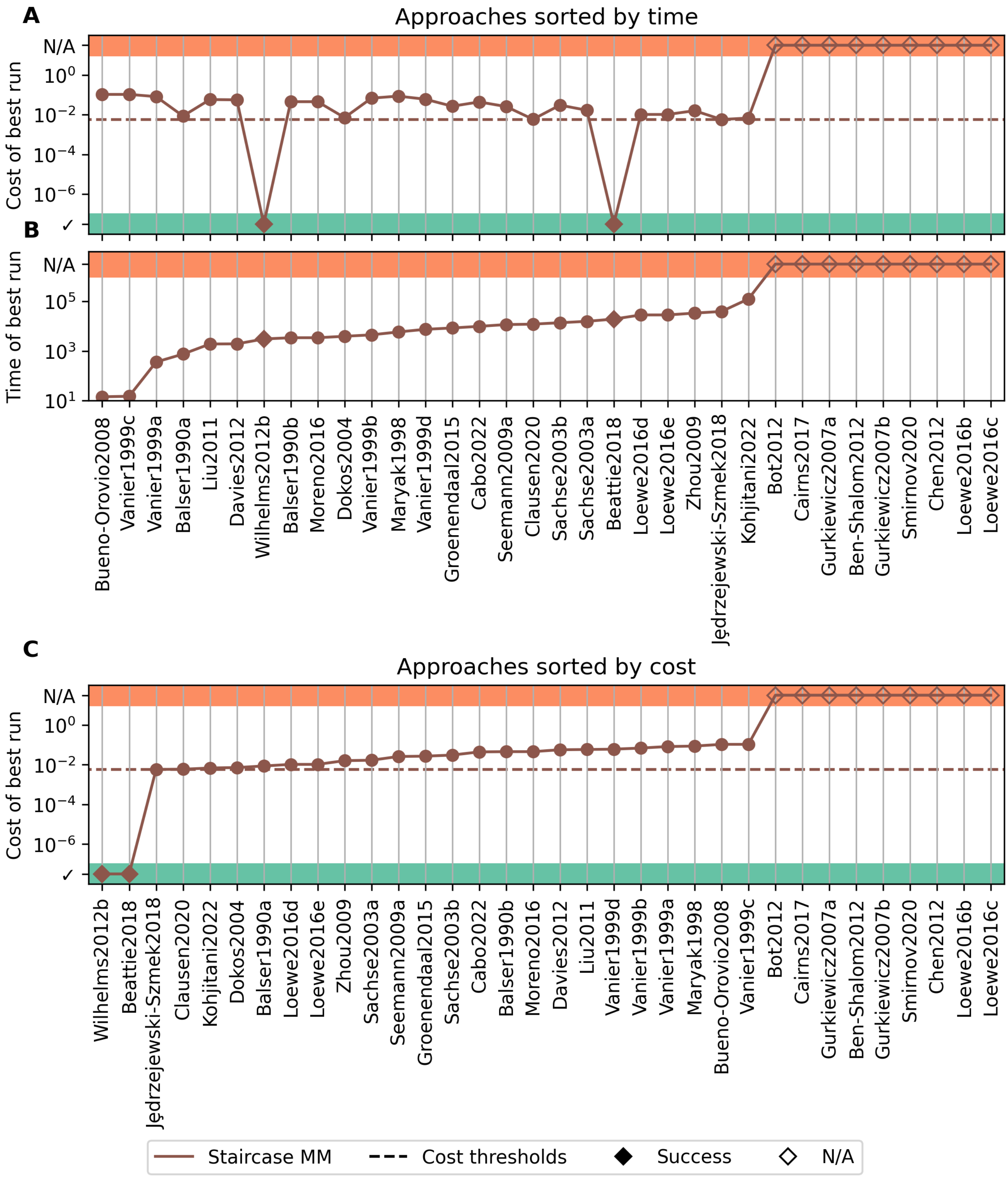

Fig K. Performance on Staircase MM. Cost or time (in FEs) of the best run for each approach on the Staircase MM problem, against approaches sorted by cost or time. (A) Cost, sorted by time. (B) Time, sorted by time. (C) Cost, sorted by cost. Cost thresholds are given as dashed lines. Successful runs are denoted by ✓; and incomplete runs by N/A.

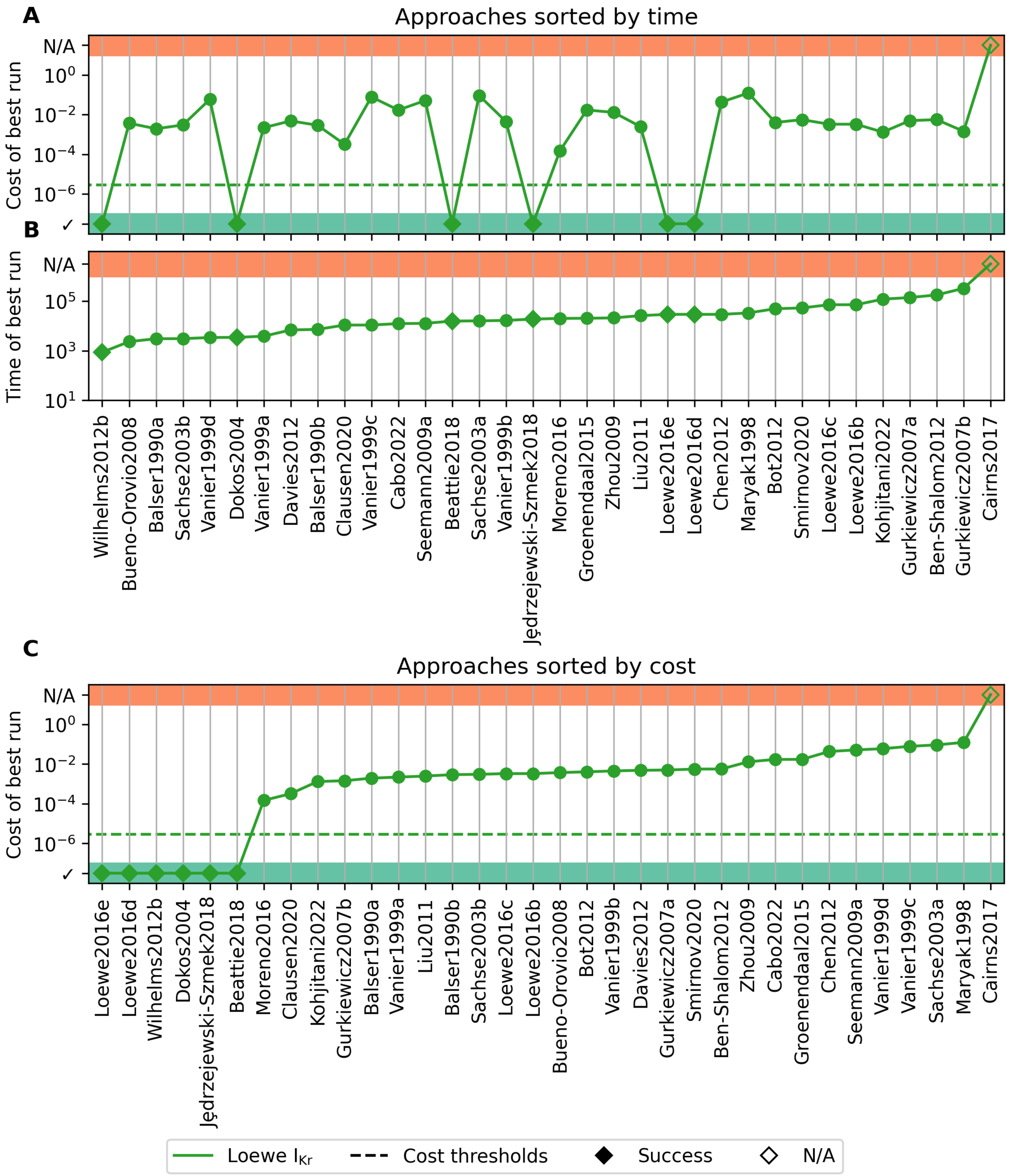

Fig L. Performance on Loewe I_{Kr}. Cost or time (in FEs) of the best run for each approach on the Loewe I_{Kr} problem, against approaches sorted by cost or time. (A) Cost, sorted by time. (B) Time, sorted by time. (C) Cost, sorted by cost. Cost thresholds are given as dashed lines. Successful runs are denoted by ✓; and incomplete runs by N/A.

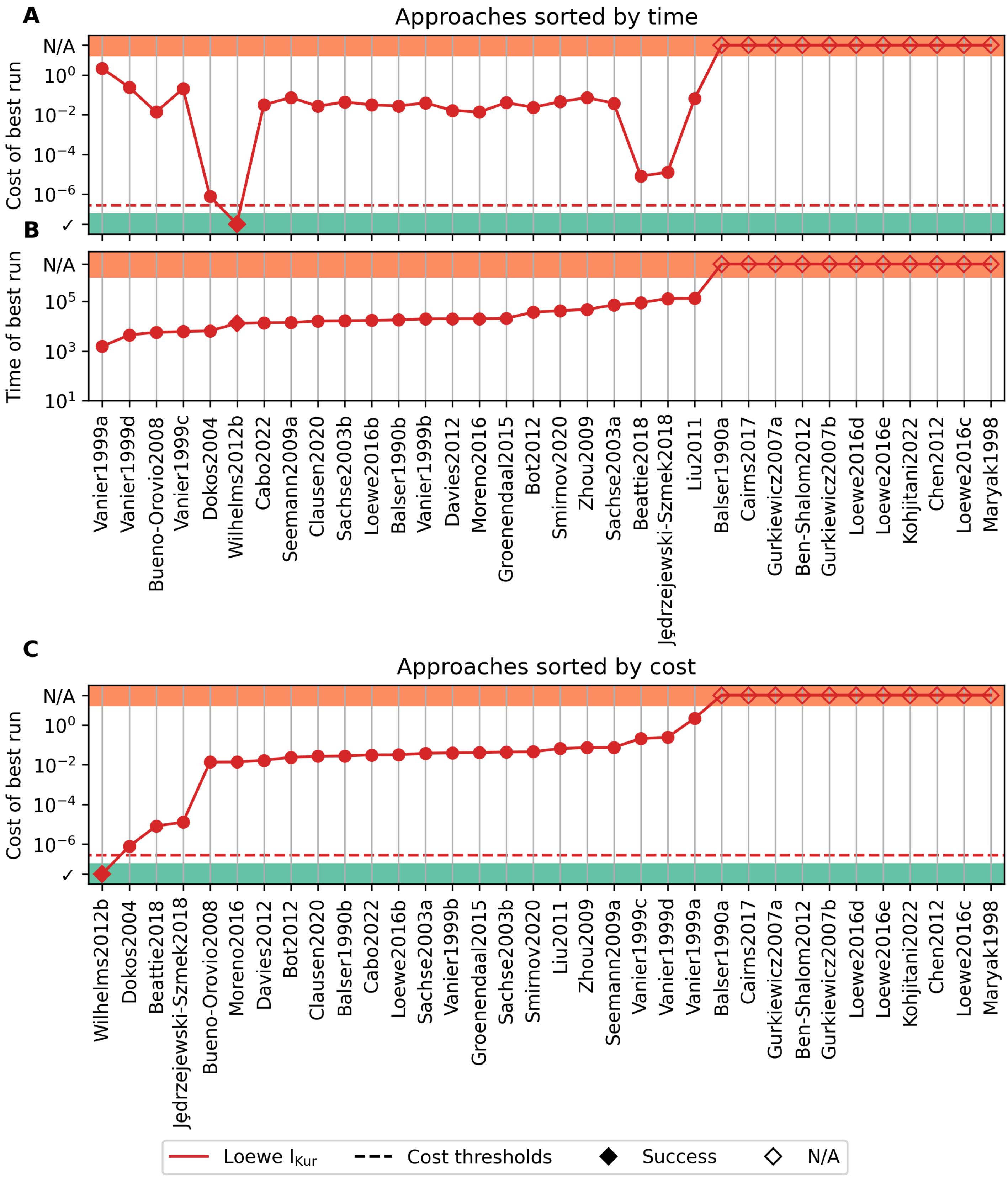

Fig M. Performance on Loewe I_{Kur}. Cost or time (in FEs) of the best run for each approach on the Loewe I_{Kur} problem, against approaches sorted by cost or time. (A) Cost, sorted by time. (B) Time, sorted by time. (C) Cost, sorted by cost. Cost thresholds are given as dashed lines. Successful runs are denoted by ✓; and incomplete runs by N/A.

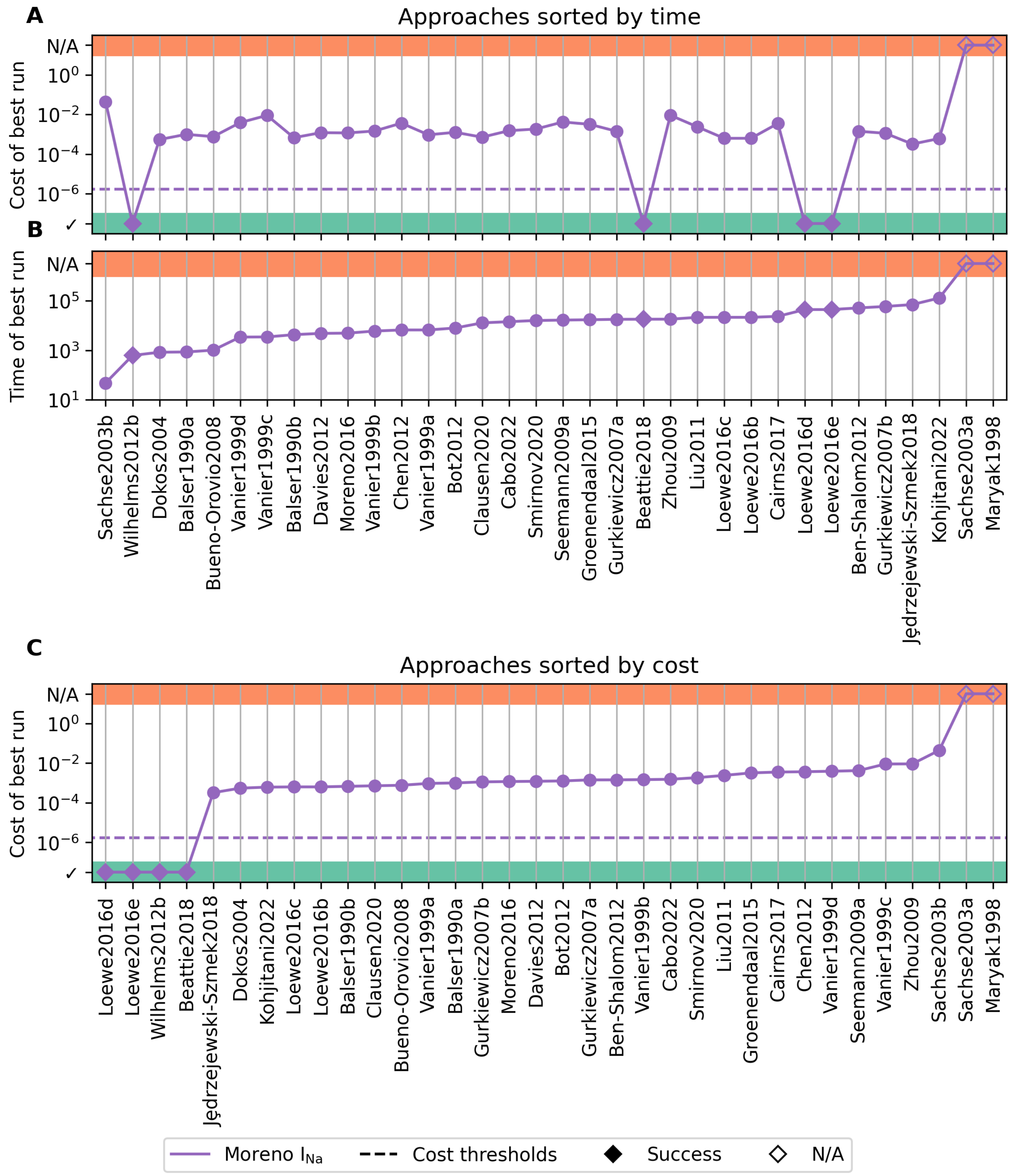

Fig N. Performance on Moreno I_{Na}. Cost or time (in FEs) of the best run for each approach on the Moreno I_{Na} problem, against approaches sorted by cost or time. (A) Cost, sorted by time. (B) Time, sorted by time. (C) Cost, sorted by cost. Cost thresholds are given as dashed lines. Successful runs are denoted by ✓; and incomplete runs by N/A.

References

1. Smirnov D, Pikunov A, Syunyaev R, Deviatiiarov R, Gusev O, Aras K, et al. Genetic algorithm-based personalized models of human cardiac action potential. PLOS ONE. 2020;15(5):e0231695. doi:10.1371/journal.pone.0231695.

2. Vanier MC, Bower JM. A comparative survey of automated parameter-search methods for compartmental neural models. Journal of Computational Neuroscience. 1999;7(2):149–171. doi:10.1023/A:1008972005316.

3. Loewe A, Wilhelms M, Schmid J, Krause MJ, Fischer F, Thomas D, et al. Parameter estimation of ion current formulations requires hybrid optimization approach to be both accurate and reliable. Frontiers in Bioengineering and Biotechnology. 2016;3:e209. doi:10.3389/fbioe.2015.00209.

4. Kreutz C. Guidelines for benchmarking of optimization-based approaches for fitting mathematical models. Genome Biology. 2019;20(1):e281. doi:10.1186/s13059-019-1887-9.

5. Wei Y, Clerx M, Mirams GR. Where's Ben Nevis? A 2D optimisation benchmark with 957,174 local optima based on Great Britain terrain data; 2024. Available from: https://arxiv.org/abs/2410.02422v1.

6. Villaverde AF, Fröhlich F, Weindl D, Hasenauer J, Banga JR. Benchmarking optimization methods for parameter estimation in large kinetic models. Bioinformatics. 2019;35(5):830–838. doi:10.1093/bioinformatics/bty736.

7. Auger A, Hansen N. A restart CMA evolution strategy with increasing population size. In: 2005 IEEE Congress on Evolutionary Computation. vol. 2. Edinburgh, UK: IEEE; 2005. p. 1769–1776.

8. Hansen N, Auger A, Ros R, Finck S, Pošík P. Comparing results of 31 algorithms from the Black-Box Optimization Benchmarking BBOB-2009. In: Proceedings of the 12th Annual Conference Companion on Genetic and Evolutionary Computation. GECCO '10. Portland, United States: Association for Computing Machinery; 2010. p. 1689–1696.

9. Hansen N, Auger A, Ros R, Mersmann O, Tušar T, Brockhoff D. COCO: a platform for comparing continuous optimizers in a black-box setting. Optimization Methods and Software. 2021;36(1):114–144. doi:10.1080/10556788.2020.1808977.

10. Fink M, Noble D. Markov models for ion channels: versatility versus identifiability and speed. Philosophical Transactions Series A, Mathematical, Physical, and Engineering Sciences. 2009;367(1896):2161–2179. doi:10.1098/rsta.2008.0301.

11. Whittaker DG, Clerx M, Lei CL, Christini DJ, Mirams GR. Calibration of ionic and cellular cardiac electrophysiology models. WIREs Systems Biology and Medicine. 2020;12(4):e1482. doi:10.1002/wsbm.1482.

12. Clerx M, Beattie KA, Gavaghan DJ, Mirams GR. Four ways to fit an ion channel model. Biophysical Journal. 2019;117(12):2420–2437. doi:10.1016/j.bpj.2019.08.001.

13. Creswell R, Shepherd KM, Lambert B, Mirams GR, Lei CL, Tavener S, et al. Understanding the impact of numerical solvers on inference for differential equation models. Journal of The Royal Society Interface. 2024;21(212):e20230369. doi:10.1098/rsif.2023.0369.

14. Nelder JA, Mead R. A simplex method for function minimization. The Computer Journal. 1965;7(4):308–313. doi:10.1093/comjnl/7.4.308.

15. Branch MA, Coleman TF, Li Y. A subspace, interior, and conjugate gradient method for large-scale bound-constrained minimization problems. SIAM Journal on Scientific Computing. 1999;21(1):1–23. doi:10.1137/S1064827595289108.

16. Hansen N, Ostermeier A. Completely derandomized self-adaptation in evolution strategies. Evolutionary Computation. 2001;9(2):159–195. doi:10.1162/106365601750190398.

17. Kennedy J, Eberhart R. Particle swarm optimization. In: Proceedings of ICNN'95 - International Conference on Neural Networks. vol. 4. Perth, WA, Australia: IEEE; 1995. p. 1942–1948.

18. Song J, Kim YJ, Leem CH. Improving the hERG model fitting using a deep learning-based method. Frontiers in Physiology. 2023;14:e1111967. doi:10.3389/fphys.2023.1111967.

19. Balser JR, Roden DM, Bennett PB. Global parameter optimization for cardiac potassium channel gating models. Biophysical Journal. 1990;57(3):433–444. doi:10.1016/S0006-3495(90)82560-1.

20. Maryak JL, Smith RH, Winslow RL. Modeling cardiac ion channel conductivity: model fitting via simulation. In: 1998 Winter Simulation Conference. Proceedings (Cat. No.98CH36274). vol. 2. Washington, DC, USA: IEEE; 1998. p. 1587–1590.

21. Clancy CE, Rudy Y. Linking a genetic defect to its cellular phenotype in a cardiac arrhythmia. Nature. 1999;400(6744):566–569. doi:10.1038/23034.

22. Sachse FB, Seemann G, Chaisaowong K, Mohr MB. Modeling of electro-mechanics of human cardiac myocytes: parameterization with numerical minimization techniques. In: Proceedings of the 25th Annual International Conference of the IEEE Engineering in Medicine and Biology Society (IEEE Cat. No.03CH37439). vol. 3. Cancun, Mexico: IEEE; 2003. p. 2810–2813.

23. Dokos S, Lovell NH. Parameter estimation in cardiac ionic models. Progress in Biophysics and Molecular Biology. 2004;85(2-3):407–431. doi:10.1016/j.pbiomolbio.2004.02.002.

24. Gurkiewicz M, Korngreen A. A numerical approach to ion channel modelling using whole-cell voltage-clamp recordings and a genetic algorithm. PLOS Computational Biology. 2007;3(8):e169. doi:10.1371/journal.pcbi.0030169.

25. Bueno-Orovio A, Cherry EM, Fenton FH. Minimal model for human ventricular action potentials in tissue. Journal of Theoretical Biology. 2008;253(3):544–560. doi:10.1016/j.jtbi.2008.03.029.

26. Seemann G, Lurz S, Keller DUJ, Weiss DL, Scholz EP, Dössel O. Adaption of mathematical ion channel models to measured data using the particle swarm optimization. In: Magjarevic R, Nagel JH, Vander Sloten J, Verdonck P, Nyssen M, Haueisen J, editors. 4th European Conference of the International Federation for Medical and Biological Engineering. vol. 22. Berlin, Heidelberg: Springer; 2009. p. 2507–2510.

27. Zhou Q, Zygmunt AC, Cordeiro JM, Siso-Nadal F, Miller RE, Buzzard GT, et al. Identification of IKr kinetics and drug binding in native myocytes. Annals of Biomedical Engineering. 2009;37(7):1294–1309. doi:10.1007/s10439-009-9690-5.

28. Guo T, Abed AA, Lovell NH, Dokos S. A generic ionic model of cardiac action potentials. In: 2010 Annual International Conference of the IEEE Engineering in Medicine and Biology. Buenos Aires, Argentina: IEEE; 2010. p. 1465–1468.

29. Liu F, Walmsley J, Burrage K. Parameter estimation for a phenomenological model of the cardiac action potential. In: McLean W, Roberts AJ, editors. Proceedings of the 15th Biennial Computational Techniques and Applications Conference, CTAC-2010. vol. 52 of ANZIAM J. New South Wales, Australia: Cambrdige University Press; 2011. p. C482–C499.

30. Ben-Shalom R, Aviv A, Razon B, Korngreen A. Optimizing ion channel models using a parallel genetic algorithm on graphical processors. Journal of Neuroscience Methods. 2012;206(2):183–194. doi:10.1016/j.jneumeth.2012.02.024.

31. Bot CT, Kherlopian AR, Ortega FA, Christini DJ, Krogh-Madsen T. Rapid genetic algorithm optimization of a mouse computational model: benefits for anthropomorphization of neonatal mouse cardiomyocytes. Frontiers in Physiology. 2012;3:e421. doi:10.3389/fphys.2012.00421.

32. Chen F, Chu A, Yang X, Lei Y, Chu J. Identification of the parameters of the Beeler–Reuter ionic equation with a partially perturbed particle swarm optimization. IEEE Transactions on Biomedical Engineering. 2012;59(12):3412–3421. doi:10.1109/TBME.2012.2216265.

33. Davies MR, Mistry HB, Hussein L, Pollard CE, Valentin JP, Swinton J, et al. An in silico canine cardiac midmyocardial action potential duration model as a tool for early drug safety assessment. American Journal of Physiology Heart and Circulatory Physiology. 2012;302(7):H1466–H1480. doi:10.1152/ajpheart.00808.2011.

34. Wilhelms M, Schmid J, Krause MJ, Konrad N, Maier J, Scholz EP, et al. Calibration of human cardiac ion current models to patch clamp measurement data. In: 2012 Computing in Cardiology; 2012. p. 229–232.

35. Al Abed A, Guo T, Lovell NH, Dokos S. Optimisation of ionic models to fit tissue action potentials: application to 3D atrial modelling. Computational and Mathematical Methods in Medicine. 2013;2013:e951234. doi:10.1155/2013/951234.

36. Du D, Yang H, Norring SA, Bennett ES. In-silico modeling of glycosylation modulation dynamics in hERG ion channels and cardiac electrical signals. IEEE Journal of Biomedical and Health Informatics. 2014;18(1):205–214. doi:10.1109/JBHI.2013.2260864.

37. Groenendaal W, Ortega FA, Kherlopian AR, Zygmunt AC, Krogh-Madsen T, Christini DJ. Cell-specific cardiac electrophysiology models. PLOS Computational Biology. 2015;11(4):e1004242. doi:10.1371/journal.pcbi.1004242.

38. Moreno JD, Lewis TJ, Clancy CE. Parameterization for in-silico modeling of ion channel interactions with drugs. PLOS ONE. 2016;11(3):e0150761. doi:10.1371/journal.pone.0150761.

39. Cairns DI, Fenton FH, Cherry EM. Efficient parameterization of cardiac action potential models using a genetic algorithm. Chaos. 2017;27(9):e093922. doi:10.1063/1.5000354.

40. Jędrzejewski-Szmek Z, Abrahao KP, Jędrzejewska-Szmek J, Lovinger DM, Blackwell KT. Parameter optimization using covariance matrix adaptation—evolutionary strategy (CMA-ES), an approach to investigate differences in channel properties between neuron subtypes. Frontiers in Neuroinformatics. 2018;12:e47. doi:10.3389/fninf.2018.00047.

41. Beattie KA, Hill AP, Bardenet R, Cui Y, Vandenberg JI, Gavaghan DJ, et al. Sinusoidal voltage protocols for rapid characterisation of ion channel kinetics. The Journal of Physiology. 2018;596(10):1813–1828. doi:10.1113/JP275733.

42. Voldsgaard Clausen M. Obtaining transition rates from single-channel data without initial parameter seeding. Channels. 2020;14(1):87–97. doi:10.1080/19336950.2020.1732004.

43. Cabo C. Positive rate-dependent action potential prolongation by modulating potassium ion channels. Physiological Reports. 2022;10(12):e15356. doi:10.14814/phy2.15356.

44. Kohjitani H, Koda S, Himeno Y, Makiyama T, Yamamoto Y, Yoshinaga D, et al. Gradient-based parameter optimization method to determine membrane ionic current composition in human induced pluripotent stem cell-derived cardiomyocytes. Scientific Reports. 2022;12(1):e19110. doi:10.1038/s41598-022-23398-0.

45. Lei CL, Clerx M, Gavaghan DJ, Polonchuk L, Mirams GR, Wang K. Rapid characterization of hERG channel kinetics I: using an automated high-throughput system. Biophysical Journal. 2019;117(12):2438–2454. doi:10.1016/j.bpj.2019.07.029.

46. Fink M, Noble D, Virag L, Varro A, Giles WR. Contributions of hERG K+ current to repolarization of the human ventricular action potential. Progress in Biophysics and Molecular Biology. 2008;96(1-3):357–376. doi:10.1016/j.pbiomolbio.2007.07.011.

47. Courtemanche M, Ramirez RJ, Nattel S. Ionic mechanisms underlying human atrial action potential properties: insights from a mathematical model. The American Journal of Physiology. 1998;275(1):H301–H321. doi:10.1152/ajpheart.1998.275.1.H301.

48. Clerx M, Collins P, de Lange E, Volders PGA. Myokit: a simple interface to cardiac cellular electrophysiology. Progress in Biophysics and Molecular Biology. 2016;120(1-3):100–114. doi:10.1016/j.pbiomolbio.2015.12.008.

49. Hindmarsh AC, Brown PN, Grant KE, Lee SL, Serban R, Shumaker DE, et al. SUNDIALS: suite of nonlinear and differential/algebraic equation solvers. ACM Transactions on Mathematical Software. 2005;31(3):363–396. doi:10.1145/1089014.1089020.

50. Gardner DJ, Reynolds DR, Woodward CS, Balos CJ. Enabling new flexibility in the SUNDIALS suite of nonlinear and differential/algebraic equation solvers. ACM Transactions on Mathematical Software. 2022;48(3):e31. doi:10.1145/3539801.

51. Rush S, Larsen H. A practical algorithm for solving dynamic membrane equations. IEEE Transactions on Biomedical Engineering. 1978;25(4):389–392. doi:10.1109/TBME.1978.326270.

52. Serban R, Hindmarsh AC. CVODES: the sensitivity-enabled ODE solver in SUNDIALS. In: ASME 2005 International Design Engineering Technical Conferences and Computers and Information in Engineering Conference. vol. 6. Long Beach, California, USA: ASME; 2005. p. 257–269.

53. Virtanen P, Gommers R, Oliphant TE, Haberland M, Reddy T, Cournapeau D, et al. SciPy 1.0: fundamental algorithms for scientific computing in Python. Nature Methods. 2020;17(3):261–272. doi:10.1038/s41592-019-0686-2.

54. Clerx M, Robinson M, Lambert B, Lei CL, Ghosh S, Mirams GR, et al. Probabilistic inference on noisy time series (PINTS). Journal of Open Research Software. 2019;7(1):e23. doi:10.5334/jors.252.

55. Blank J, Deb K. Pymoo: multi-objective optimization in Python. IEEE Access. 2020;8:89497–89509. doi:10.1109/ACCESS.2020.2990567.

56. Auger A, Hansen N. Performance evaluation of an advanced local search evolutionary algorithm. In: 2005 IEEE Congress on Evolutionary Computation. vol. 2. Edinburgh, UK: IEEE; 2005. p. 1777–1784.

57. Kreutz C. New concepts for evaluating the performance of computational methods*. IFAC-PapersOnLine. 2016;49(26):63–70. doi:10.1016/j.ifacol.2016.12.104.

58. Hass H, Loos C, Raimúndez-Álvarez E, Timmer J, Hasenauer J, Kreutz C. Benchmark problems for dynamic modeling of intracellular processes. Bioinformatics. 2019;35(17):3073–3082. doi:10.1093/bioinformatics/btz020.

59. Nogaret A. Approaches to parameter estimation from model neurons and biological neurons. Algorithms. 2022;15(5):e168. doi:10.3390/a15050168.

60. Egea JA, Balsa-Canto E, García MSG, Banga JR. Dynamic optimization of nonlinear processes with an enhanced scatter search method. Industrial & Engineering Chemistry Research. 2009;48(9):4388–4401. doi:10.1021/ie801717t.

61. Whittaker DG, Wang J, Shuttleworth JG, Venkateshappa R, Kemp JM, Claydon TW, et al. Ion channel model reduction using manifold boundaries. Journal of The Royal Society Interface. 2022;19(193):20220193. doi:10.1098/rsif.2022.0193.

62. Mangold KE, Wang W, Johnson EK, Bhagavan D, Moreno JD, Nerbonne JM, et al. Identification of structures for ion channel kinetic models. PLOS Computational Biology. 2021;17(8):e1008932. doi:10.1371/journal.pcbi.1008932.

63. Shuttleworth JG, Lei CL, Whittaker DG, Windley MJ, Hill AP, Preston SP, et al. Empirical quantification of predictive uncertainty due to model discrepancy by training with an ensemble of experimental designs: an application to ion channel kinetics. Bulletin of Mathematical Biology. 2023;86(1):e2. doi:10.1007/s11538-023-01224-6.

64. Lei CL, Clerx M, Gavaghan DJ, Mirams GR. Model-driven optimal experimental design for calibrating cardiac electrophysiology models. Computer Methods and Programs in Biomedicine. 2023;240:e107690. doi:10.1016/j.cmpb.2023.107690.
