# Supplementary material for: IonBench: A benchmark of optimisation strategies for mathematical models of ion channel currents
Source: PLoS Comput Biol. 2025 Aug 14;21(8):e1013319. doi: 10.1371/journal.pcbi.1013319 (PMC12373289; doi:10.1371/journal.pcbi.1013319)
Supplement: Attachment — Submitted filename: Response to Reviewers.pdf [file pcbi.1013319.s002.pdf]

Responses to reviewers for “IonBench: a benchmark of optimisation strategies for mathematical models of ion channel currents”

Reviewer #1

1. Lines 157ff: The choice of benchmark problems is not obvious
We have now expanded on the choice of benchmark problems and given the reasons behind these choices (lines 158ff, 198ff and Table 2).
2. What's the rationale behind choosing those 4 specific problems?
In summary, the benchmark problems were chosen to cover all of the typical challenges faced during optimization of cardiac ion channels. The current problems provide a small set such that there is good variety in all outlined challenges. This is now made clear in the manuscript (lines 158ff, 198ff and Table 2).
3. Why were problems 1-4 evaluated by comparing currents whereas 5 was compared in terms of summary statistics? (line 176)
While it is beneficial to fit to current traces, full current traces are not always available. As such, it is important to understand how these methods perform when postprocessing is performed on the data, and whether any of the methods differ in how well they handle the changes to the fitness landscape given by the postprocessing.
4. Noise was added to benchmark problems 1+2 but not to 3-5. Why? Would you suspect the results to change if noise was present (as is inevitably the case in any real-world application of these methods)? Previous studies reported that small levels of noise can even lead to better results for gradient-based optimization whereas performance gets worse for higher noise levels.
While noise will be included in any real world application, these approaches are commonly tested without noise, since it makes interpretation of the cost easier (a RMSE-based cost has a global minimum of 0 at the parameters that generated the data). For this reason we wanted problems both with and without noise. In order to keep the problem set small, we did not explore different noise strengths. We have included a section in the discussion on different noise strengths and how it could affect the optimisation approaches differently (lines 621ff).
5. The versatility of the provided code could be increased by exposing the level of noise as a configurable parameter.
We have now added the noise strength as a configurable parameter in ionBench v1.0.

6. Why are the parameter sampling regions for problem 5 smaller (+/-25%) than for the other problems?
The size of the parameter sampling region was predefined in Moreno et al. Smaller parameter search spaces will typically favor local optimiser approaches (since there are fewer local optima, the approach is more likely to find the global optima). Rather than wanting to know if an approach works well on smaller spaces, you may wish to know how well an approach performs on very large spaces, and including smaller spaces provides a reference against which you can compare.
7. It would be interesting to see the cost values and runtime achieved with all approaches (even if not undershooting the cost threshold considered "successful") as the small number of approaches (compared to the 34 candidates) shown in Fig. 6 is surprising.
Unfortunately, the calculation of ERT (the values reported in Fig 6), requires at least one of the attempts to be successful. While we do estimate it for unsuccessful approaches to calculate the significance, this estimate only considers the time to run the approach (and the number of times the approach was attempted), and does not consider the cost achieved by the approach (only whether or not it was successful). Fig 7 shows the cost and runtime of all approaches (specifically their best performing/lowest cost run). As given in response 1.13, we have included some more supplemental versions of Fig 7 for each problem, as well as a table of the data to make it more readable (Supplemental E).
8. The suggested optimal algorithm is a multi-start approach. Some guidance on the number of required starts would be helpful for future users of this approach as the achievable cost in real-world scenarios will be markedly above 0.
We have included some guidance on the number of restarts in the discussion (lines 565ff).
9. The relation of the described approaches with machine learning approaches for emulating the AP and for parameter optimization could be further elaborated.
We have included a section on the use of emulators and how they relate to the results (particularly in whether or not gradient calculations are possible/reliable) into the discussion (lines 595ff).
10. Fig. 2: Should "RMSE: cost" read "RMSE, cost?"
This has now been corrected to “Cost”.
11. Fig. 5 and lines 255ff: I have trouble identifying the lower bound 10^-3 in the figure. Please elaborate the caption or adapt the figure.
The figure has been adapted to show the change in penalty at 10^-3, while also showing how the penalty changes linearly on lower bound violations and logarithmically on upper bound violations.
12. Lines 297ff./404: I consider the information on the unidentifiable parameters very valuable. Please provide more guidance on how to read and interpret the plot in the supplement and consider moving key results (which ones were unidentifiable?) to the main manuscript

When decided on which parameters are unidentifiable, we are mainly focused on the use case of calculating the cost threshold for each problem. We want the cost threshold to represent a reasonable cost such that, if the cost of some point is below the cost threshold, we have likely identified all (identifiable) parameters to within 5%. Some parameters need to be excluded from this calculation because it would lead to a cost threshold that is too close to the minimum, and the ODE solver tolerances needed to ensure the cost function surface is smooth is this region would be unreasonable. Even though most of the parameters in the Staircase MM problem would be generally considered unidentifiable in other works, since they aren't completely flat, they do not need to be removed from this calculation (they effectively get removed when we round up to 3 s.f.). For this reason, which specific parameters were unidentifiable isn't relevant to the work compared with the cost thresholds.
We have expanded on the reasons for looking at parameter identifiability, and how to read the profile likelihood plots, in the Supplemental D and included some more of the results in the section "Parameter identifiability" (lines 425ff). We have clarified which parameters were removed from the calculation of the Loewe IKur cost threshold in Supplemental D.
13. Fig. 7 is a bit hard to read with intersecting lines and overlapping symbols. How about providing this information in addition in a supplementary table?
We have added the data into Supplemental E, as well as reproducing Fig 7 for each problem, again presented in Supplemental E.

Reviewer #2

1. It was somewhat surprising that so many (the vast majority) of the published methods were unable to effectively minimize the cost for any of the chosen formulations. I would ask for some discussion if this finding is consistent with the cited publications on these methods, or if it is a function of the methods chosen in their developed benchmark system? In other words, were these published methods able to solve similar problems elsewhere, but not in your benchmark?
When these approaches are evaluated, they are rarely tested against a particular cost threshold, especially ones as strict as we use. This leads to the approaches being able to reproduce the data approximately (which can be done around costs of 10^-2 for the problems without noise, which most approaches do achieve in Fig 7), but aren't able to utilise all the information in the data and get close to identifying the data generating parameters (which requires costs closer to the cost threshold). We have added a section to the discussion on this (lines 533ff).
2. The choice of model set-up could be better discussed, in particular the choice of noise. Why was this specific level of noise added, and why to only a few of the model formulations? The choice seems a little arbitrary, and yet could potentially be having a strong effect on the results.

We have now included a description of how this particular problem set was derived (lines 158ff, 198ff, and Table 2). We wished to include both problems with and without noise (as many approaches are evaluated in a noise-free environment) in order to allow investigation of how the presence of noise alters the performance of the different approaches (this didn't end up being necessary due to the consistency of the Wilhelms2012b approach).
We did not investigate how the strength of the noise affects performance in order to keep the problem set small. Although, now that a smaller set of sensible approaches has been identified, a more thorough search of a much larger problem set would be possible.
We have included in the discussion some comments around how the choice of noise level can affect the optimisation approaches differently (lines 621ff).
3. All benchmarks are shown in terms of cost and time. I think the results would be well supplemented with additionally showing parameter error, as this in the end is the actual goal of this type of optimization process. This obviously could show poor results in the case where there are so many parameters that are unidentifiable, but I think the reader can contextualize it sufficiently.
During development we did consider using parameter error but it comes with some problems that we do not feel are recoverable:

- As you pointed out, unidentifiability can lead to arbitrarily far away parameters producing the same cost. Since it is not possible for an approach to differentiate between these points, this ends up randomly penalising some approaches because of where they ended up, requiring many more optimisations to accurately assess if one approach is better than another;
- Since the introduction of noise can shift the minimum (as was the case for the Staircase MM problem), it can be possible to find a lower cost with high parameter error. It would be unfair to penalise an approach for finding a lower cost - especially when all approaches will identify this "low cost - high parameter error" point as best given sufficient compute time/number of runs;
- Since the approaches only ever have access to the cost function surface, looking at parameter error doesn't provide any additional value (unidentifiable problems will have higher and more unpredictable parameter error but the underlying results for the approaches remains the same).

Since low parameter error is always the true goal, we have added some points to the discussion on this. In particular, how to best align minimising the cost function with minimising parameter error (lines 640ff).
4. In the profile curves, I was uncertain of the difference between the 'optimized' and 'unoptimized' profiles.
The "optimized" curves are typical profile likelihood curves, where the likelihood (or cost in our case) is optimized over the parameters, keeping one parameter (the x axis parameter) fixed at some value (given by the x axis). The unoptimized profiles do not perform this optimization, keeping all parameters apart from the x axis parameter at its default value. This gives a slice of the cost function through the x

axis, to provide a scale for the cost function surface against the “optimized” profile likelihood curves.
We have now added this description to Supplemental D.
